# Herpes Zoster Risk Reduction through Exposure to Chickenpox Patients: A Systematic Multidisciplinary Review

**DOI:** 10.1371/journal.pone.0066485

**Published:** 2013-06-21

**Authors:** Benson Ogunjimi, Pierre Van Damme, Philippe Beutels

**Affiliations:** 1 Centre for Health Economics Research and Modeling Infectious Diseases, Vaccine and Infectious Disease Institute, University of Antwerp, Antwerp, Belgium; 2 Interuniversity Institute for Biostatistics and Statistical Bioinformatics, Hasselt University, Hasselt, Belgium; 3 Centre for the Evaluation of Vaccination, Vaccine and Infectious Disease Institute, University of Antwerp, Antwerp, Belgium; 4 School of Public Health and Community Medicine, University of New South Wales, Sydney, Australia; National Institute for Public Health and the Environment, The Netherlands

## Abstract

Varicella-zoster virus (VZV) causes chickenpox and may subsequently reactivate to cause herpes zoster later in life. The exogenous boosting hypothesis states that re-exposure to circulating VZV can inhibit VZV reactivation and consequently also herpes zoster in VZV-immune individuals. Using this hypothesis, mathematical models predicted widespread chickenpox vaccination to increase herpes zoster incidence over more than 30 years. Some countries have postponed universal chickenpox vaccination, at least partially based on this prediction. After a systematic search and selection procedure, we analyzed different types of exogenous boosting studies. We graded 13 observational studies on herpes zoster incidence after widespread chickenpox vaccination, 4 longitudinal studies on VZV immunity after re-exposure, 9 epidemiological risk factor studies, 7 mathematical modeling studies as well as 7 other studies. We conclude that exogenous boosting exists, although not for all persons, nor in all situations. Its magnitude is yet to be determined adequately in any study field.

## Introduction

Primary infection by varicella-zoster virus (VZV) causes the clinical syndrome ‘chickenpox’ (CP), mainly in childhood. An effective commercial childhood CP vaccine has been available for nearly 20 years. It is recommended to be used in a two-dose schedule because experience with a single dose has led to frequent (milder) breakthrough infections [1], [2]. After primary infection VZV remains latent in neural ganglia until reactivation. Herpes zoster (HZ), also called shingles, is caused by the symptomatic reactivation of VZV and this reactivation is assumed to be a consequence of a lower cellular immunity mainly in immunocompromised or older individuals [3]–[5]. Other risk factors for HZ have been identified and include gender, ethnicity, host susceptibility and depression (see review by Thomas and Hall [6]). Compared to CP, HZ is associated with relatively higher morbidity and costs (see for e.g. Bilcke et al [7]). A vaccine against HZ exists and is shown to be effective probably due to the long-term augmentation of VZV-specific cellular immunity [8]. The efficacy of this vaccine partially supports the exogenous boosting hypothesis, although one should take the different routes of exposure (i.e. subcutaneously vs. through mucosa) into account. Hope-Simpson first postulated that re-exposure to circulating VZV could inhibit reactivation of VZV [9]. A consequence of this so-called ‘exogenous boosting’ hypothesis would be a temporary increase in HZ cases following the reduced circulation of VZV, under the influence of a universal childhood CP vaccination program. This HZ increase is expected to be temporary because of the increasing proportion of CP vaccinated individuals who are generally assumed to be no longer at risk for HZ. Several population-based mathematical modeling papers (the first of which published by Schuette and Hethcote [10]) predicted substantial HZ incidence increases in over 30 years following the introduction of widespread CP vaccination and thus called into question the overall public health impact of CP vaccination. Although several countries across the world have already implemented universal childhood CP vaccination (USA, Australia, Germany, Japan, Taiwan, Greece) many other countries continue to wait for more conclusive data regarding the existence, duration, and thus the effect of exogenous boosting. This paper presents the first systematic and multidisciplinary in-depth assessment of the literature with respect to exogenous boosting.

## Methods

### Search Background

The multidisciplinary approach of our review is focused on two primary end points: (1) HZ incidence as a function of exposure to CP and (2) the longitudinal course of VZV-specific immunity elicited by contact with CP. Both end points will depend on the type and duration of contact with CP. Other factors potentially of influence include the age of the CP patient, the age of the exposed person, environmental factors (e.g. season), the type of contact, the elapsed time between current and previous exposures, and the pre-exposure immunity level.

The results from our review are presented following the PRISMA methodology, when applicable [11].

### Search Strategy

Both PubMed and Web of Science (v5.8) were searched up to 27^th^ November 2012 without a restriction on the publication date. Our review included original research articles and letters, published at any time.

The PubMed search used the following search string (‘*’ = wildcard): (("Herpes Zoster"[Mesh] OR zoster* OR shingles OR varicella OR "chickenpox"[Mesh]) AND (exposure* OR reinfection* OR re*infection* OR boost* OR seroepidemiology OR sero-epidemiology OR "seroepidemiologic studies"[Mesh])) AND (english[la] OR English Abstract[pt]) AND (hasabstract OR letter[pt]) NOT (review[pt] OR guideline[pt] OR editorial[pt]). The Web of Science search used the following search string: Topic = (zoster OR shingles OR varicella OR chickenpox) AND Topic = (exposure OR reinfection OR boost OR seroepidemiology) with further document refinement for document types “article” or “letter” and languages “English”. This search was done with lemmatization ON (the search includes inflected forms of words in a Topic and/or Title search query, which allows for a broader scope of functionality and includes synonyms, plurals, and singulars).

**Figure 1:**
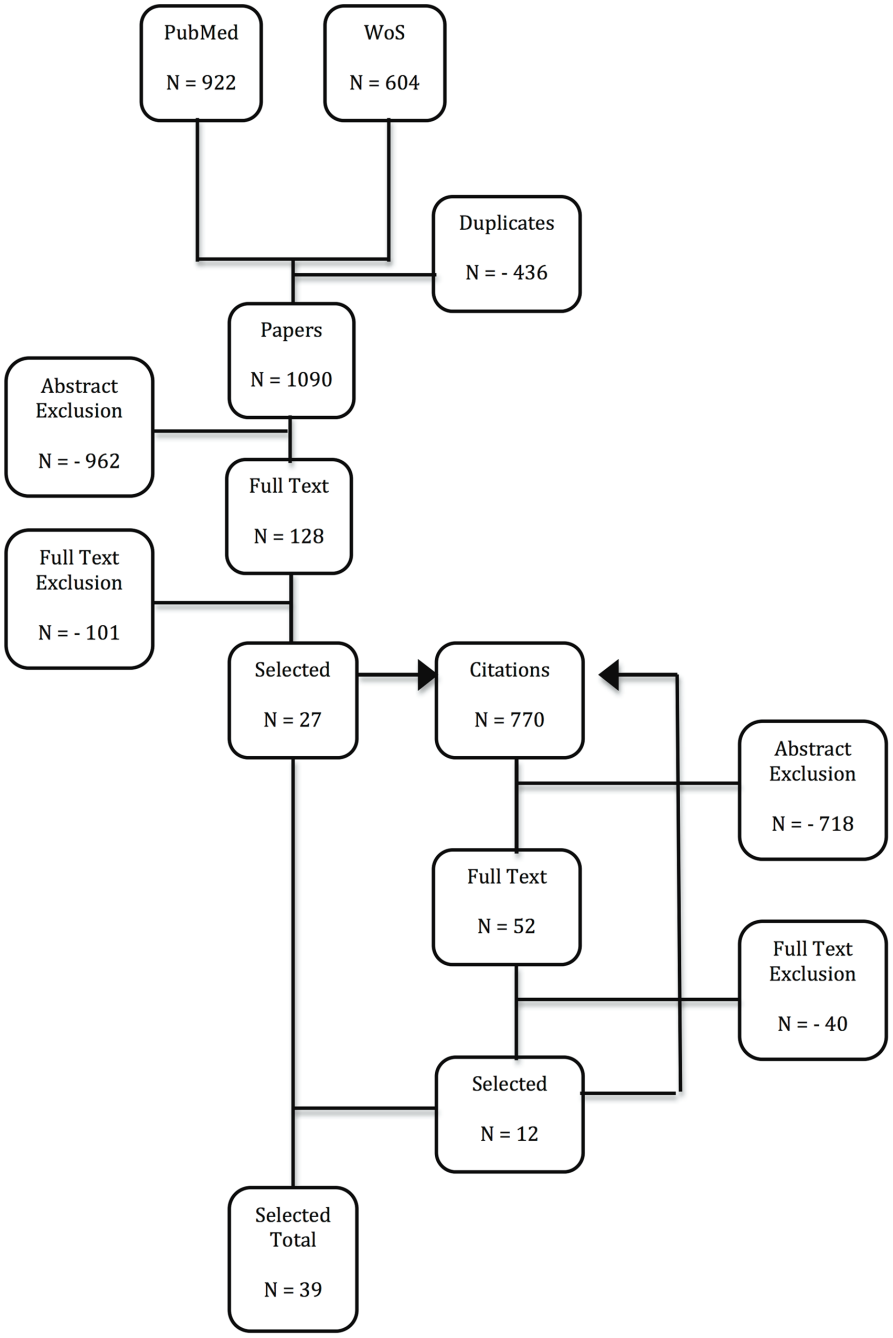
Modified PRISMA flow diagram. PubMed and Web of Science (WoS) search results were combined and after controlling for duplicates, titles and abstracts from references were screened for further full text assessment. Citations from and to the selected references were also screened using title and abstract for further full text assessment. Citations from and to the additional selected references were again screened as discussed. The additional selected references thus found were added to the earlier found references in order to obtain the Selected Total.

All search results were aggregated in Endnote X5 for MAC OS and duplicates were discarded, such that 1090 unique references were retained at first. The reference lists from these publications were assessed by screening title and abstract of references not identified in our original search. Additionally, publications citing our selections were identified and screened using Web of Science, implying published comments on our selections were also considered. That is, publications being cited by and citing the papers we retained originally, were in turn considered for additional inclusion. Citations to and by the selected publications were again screened for inclusion (see Figure 1). This way, an additional 770 publications were screened.

### Selection Criteria and Inclusion

The inclusion criteria were agreed upon by all authors and are presented in Table S1. BO first read all titles & abstracts of references from the combined PubMed and Web of Science search and organized them in 8 categories for additional assessment by PB and PVD to decide whether full text assessment was deemed suitable. All references in disagreement were further discussed until agreement was reached. The categories were: epidemiological and modeling papers included (PB) and excluded (PB), immunological or clinical papers included (PVD) and excluded (PVD), papers not related to or without a focus on VZV (PB), not original research papers (PVD) and papers without abstracts included (PB, PVD) and excluded (PB, PVD).

One hundred and twenty-eight papers were included for full text assessment and 27 papers were retained after full text assessment. The identification of 770 additional papers through citations led to the selection of 12 extra papers (see Figure 1). One non-English paper was selected for full text assessment, but could not be retrieved [12].

The methodology and results from these papers are summarized in Tables 1, 2, 3, and 4, using 5 categories: (1) 13 HZ incidence studies in countries with widespread CP vaccination, (2) 7 mathematical modeling studies, (3) 9 epidemiological risk factor studies, (4) 4 prospective longitudinal studies on VZV-immunity post-exposure and (5) 7 other studies (non-longitudinal immunological studies and an epidemiological study). We note that 40 studies are mentioned, whereas only 39 papers were included. This is caused by the Brisson et al paper [13] that had both a mathematical modeling aspect and an epidemiological risk factor aspect.

**Table 1:** Description of selected observational studies on HZ incidence in populations with a widespread chickenpox vaccination program.

Reference	Location	Time Period	Methods and data§	Main Results	Quality*	Study Design£	B**
Mullooly et al (2005)^14^	Oregon & Washington (USA)	1997–2002	Type: Retrospective database analysisData: HMO database including inpatient and outpatient HZ registrationComparison with Harvard Community Health Plan (HCHP) HZ incidence in New England (USA) during 1990–1992CP vaccination: cumulative vaccination among 2 year-olds rose from 25–35% in 1996 to 82–85% in 2002Analysis: Poisson regressionAnalysis takes changing demography into accountAnalysis takes changes in underlying diseases or immunocompromised states into account	An age and gender standardized comparison showed the 1997–2002 HZ incidence to be 27% higher than the 1990–1992 HZ incidence observed in the older HCHP database (not covering the same geographic region). The 1997–2002 HZ incidence in 0–14y was >3 x the 1990–1992 incidencePoisson regression over 1997–2002 including age, sex, age x sex, state and calendar year x age showed only a significant secular increase limited to children aged 10–17 years (RR 1.10 per calendar year, 95% CI 1.04–1.17)The authors noted that oral steroid exposure in children tended to increase in 1986–2002. Using a children-only Poisson regression model including calendar year x age and steroid exposure they found a significant effect of steroid exposure (RR 2.6), and the increase in 10–17y was no longer significant (RR 95% CI 0.91–1.21)	M	A	–
Yih et al (2005)^15^	Massachusetts (USA)	1998–2000 & 2002–2003	Type: Retrospective surveyData: random-digit-dialing to recruit adults aged >18yCP vaccination: from 1996 publicly funded and coverage increased from 23–48% in 1997–98 to 89% in 2003Analysis: Estimation of annual age-specific incidence of CP and HZAnalysis takes changing demography into account	Between 1998 and 2003 overall unadjusted CP incidence went from 16.5/1000PY to 3.5/1000PYAge-standardized HZ incidence went from 2.77/1000PY in 1999 to 5.25/1000PY in 2003 with an increasing trend (p<0.0009); the overall increase in age-standardized incidence was 90% over a 5y period; an age-group specific significant increase was seen for 25–44y (161% crude change) and >65y (70% crude change). Also a crude 152% increase in <25y (p = 0.10) was noted.	M	A	+
Jumaan et al (2005)^16^	Washington State (USA)	1992–2002	Type: Retrospective database analysisData: HMO database with CP and HZ registrationCP vaccination: coverage among children 2y olds went from <1% in 1995 to 65% in 2002Analysis: Linear-trend tests of incidence rates by use of Poisson regressionAnalysis includes pre-vaccination HZ data to compare with post-vaccination dataAnalysis takes changing demography in account	Crude incidence of CP during 1992–1998 fluctuated initially between 2.44/1000PY in 1995 to 2.00/1000PY in 1998, and then steadily decreased to 0.77/1000PY in 2002Age-standardized HZ incidence ranged between 4.05/1000PY in 1992 and 3.47/1000PY in 2000Among unvaccinated 0–9y olds HZ incidence increased from 0.87/1000PY in 1996 to 1.45/1000PY in 2002	M	A	–
Patel et al (2008)^17^	USA	1993–2004	Type: retrospective database analysisData: non-federal, short-term, general and other specialty CP and HZ hospitalization discharge ratesCP vaccination: from 1995 publicly funded, CP vaccination rates among young children were cited from CDC references to have increased from 12.2% in 1996 to 87.5% in 2004Analysis: descriptive statisticsAnalysis includes pre-vaccination HZ data to compare with post-vaccination dataAnalysis takes changing demography into account	CP related hospitalizations decreased substantially as of 1997 (500% decrease from 1993–95 to 2004)Population-adjusted HZ hospitalization rates were stable up to 2001 after which the overall rate increased up till the end of the observations, when the rate was significantly higher than the rates before 2002; an age-specific analysis showed the increase only to be present in >65y (+23% over the study period)	L	A	+
Rimland et al (2010)^18^	USA	2000–2007	Type: Retrospective database analysisData: national inpatient and outpatient HZ (primary and secondary) in veteransCP vaccination: not reported, probably similar to other USA studiesAnalysis: chi-square test for trend in total and age-specific HZ rates for entire period. HZ rates were expressed with the total number of veterans seen that year in the denominatorAnalysis takes changing demography into accountAnalysis takes a change in underlying diseases or immunocompromised states into account for HZ hospitalization	HZ total incidence increased from 3.1/1000 in 2000 to 5.22/1000 in 2007; however, only veterans older than 40y had a significant increase; in an analysis focusing on HZ cases from the Atlanta VA Medical Center, the increasing trend over time was still noted after excluding HIV, malignancies and chemotherapy; however, it is not clear whether age-standardization was applied for the latter analysis	M	A	+
Carville et al (2010)^19^	Victoria (Australia)	1995–2007	Type: Retrospective database analysisData: hospitalization & Melbourne Medical Deputising Service (MMDS): registration based on MD diagnosis during after hours medical phone callsCP vaccination: private from 2000, funded from Nov 2005, no specific vaccine uptake data reported, but distribution numbers were presentedAnalysis: Poisson or negative Binomial regressionAnalysis includes pre-vaccination HZ data to compare with post-vaccination dataAnalysis takes changing demography into accountAnalysis takes a change in underlying diseases or immunocompromised states into account for HZ hospitalization	A 4%/y decline in CP incidence from 2000 to 2007 was notedOverall HZ hospitalization increased 5%/y (95%CI 3–6%) over 1998–2007 with a main effect in 80+ year olds; HZ incidence MMDS registration increased 13%/y from 2000 to 2007; both HZ hospitalization and MMDS rates already increased in the years before the start of CP vaccination	M	A	+/−
Nelson et al (2010)^22^	Australia	1998–2009	Type: Retrospective database analysisData: GP national representative sampleCP vaccination: private from 2000, funded from Nov 2005, no specific vaccine uptake data reportedAnalysis: linear regression	CP annual decrease of 0.12 per 1000 consultations (1999–2009), most pronounced in 2005–2009Linear regression showed an annual average increase of 0.05 HZ consultations per 1000 consultations (p<0.01)	L	A	+
Grant et al (2010)^20^	Victoria (Australia)	1998–2010	Update on MMDS data from Carville et al^19^ presented as CP and HZ rate per 1000 consultationsAnalysis includes pre-vaccination HZ data to compare with post-vaccination data	Between 2000 and 2010, CP decreased from 3.3 to 1.0 per 1000 consultations and HZ increased from 1.7 to 3.4 per 1000 consultations; the HZ increase was most prominent in 80+ year olds but an increasing trend in HZ already occurred in this group before the major CP decrease in 2005–2006	L	A	+
Jardine et al (2010)^24^	Australia	1998–2009	Type: Retrospective database analysisData: hospitalization (1998–2007), ER (only for New South Wales, 2001–2009) & acyclovir (& derivatives) use (11/05- 03/09)CP vaccination: private from 1999, funded from Nov 2005, no specific vaccine coverage data were reported, CP incidence can be inferred from Nelson et al.^22^Analysis: univariate Poisson regression for hospitalization data, multivariate Poisson for ER and medicationAnalysis includes pre-vaccination HZ data to compare with post-vaccination dataAnalysis takes changing demography in account	HZ Hospitalization data showed no longitudinal changein <20y and 60+ and a decrease in 20–59y; HZ ER data showed increases of 2%-5.7% per year except for the age group<20y (non-significant increase of 1.4%); antiviral use increased in all age groups (1.7–3%/y) except in <20y	L	A	+
Carville et al (2011)^21^	Victoria (Australia)	2000–2010	Update on MMDS data from Carville et al^19^ presented as HZ incidenceAnalysis takes changing demography in account	HZ incidence increase was noted in all age groups; incidence increased 8%/Y in 70–79y and 80+ and 5%/Y in 60–69y	L	A	+
Tanuseputro et al (2011)^25^	Ontario (Canada)	1992–2010	Type: Retrospective database analysisData: hospitalizations, visits to physician offices and ERCP vaccination: private between 1999–2004 private, from 2005 publicly funded; CP vaccine sales, but not coverage data were reportedAnalysis: Taylor series confidence intervalsAnalysis includes pre-vaccination HZ data to compare with post-vaccination dataAnalysis takes changing demography into account	Moderate HZ incidence increase in last year for age groups 60+ (no statistics), but possibly due to miscoding for HZ vaccination	M	A	–
Leung et al (2011)^26^	USA	1993–2006	Type: Retrospective database analysisData: Marketscan database (private and public)CP vaccination: CP vaccination licensed from 1995; 12 states with lower CP coverage and 13 states with higher coverage than the US national median coverage during each year from 1997–2006Analysis: Control for secular changes in health care access by including 10 other conditions, also control for immunosuppression by subgroup analysisGeneralized linear modeling approachAnalysis includes pre-vaccination HZ data to compare with post-vaccination dataAnalysis takes changing demography in accountAnalysis takes a change in underlying diseases or immunocompromised states in account	An age-standardized 98% HZ incidence increase was noted over the 13y period and an increase remained present when only focusing on immunocompetent individuals (factor 1.3–1.4 increase in age-standardized rate over 8y); however, HZ incidence increases were already detected in 1993–1996 (p<0.001) and age-specific HZ incidence was the same for adults living in states with high varicella vaccine coverage and those in low-coverage states (p = 0.3173), although it was difficult to assess the value of the observation since no specific information on the actual differences in CP vaccine uptake or CP incidence between high and low vaccine coverage states was shownAdults 20–50y with dependents aged <12y initially had lower HZ incidence compared with adults without dependents (P<0.01); importantly, though the HZ incidence increased for both groups, it increased significantly more in those with dependent children, such that HZ incidence in both groups completely converged over time	H	A	+/−
Chao et al (2011)^27^	Taiwan	2000–2008	Type: Retrospective database analysisData: outpatient CP and HZ incidence dataCP vaccination: private from 1997, funded from 1998 in Taipei, from 1999 in Taichung city and nationwide from 2004 (abrupt increase to 80% coverage in 2004, increasing further to 94%)Analysis: non-parametric Page’s trend test to examine the monotony of the trends, chi-square to compare areas and Poisson multiple regression with age-gender standardizationAnalysis includes pre-vaccination HZ data to compare with post-vaccination dataAnalysis takes changing demography into accountAnalysis takes a change in comorbidities into account	The crude CP rate showed an overall decreasing trend (factor 3.7) from 2000 to 2008. Also a 20% increase in comorbidities was noted from 2000 to 2008After controlling for confounding variables such as age, gender, and comorbidities the period 2006–2008 showed an overall HZ increase of 20% compared to 2004–2008, but the increase was already noted before national introduction of CP vaccination; the age-standardized HZ incidence in Taiwan increased by 18% in 2000–2008 (in particular for 50+); however, in 70–79y there was a reduction in 2008 and for >80+ the HZ incidence peak was reached in 2004 before decreasing minimally (possibly due to small numbers)	H	A	+

*HMO* health maintenance organization; *HZ* herpes zoster; *CP* chickenpox; *RR* relative risk; *PY* person-years; *MMDS* Melbourne Medical Deputising Service; *ER* emergency room.

§ Descriptions of vaccination uptake are as reported in the respective original papers.

* H = High: the quality of methods used in this paper permits the results, within the scope of the study design, to be interpreted with at the most a few remarks. M = Medium: the quality of methods used in this paper permits the results, within the scope of the study design, to be interpreted, but with some caution. L = Low: the quality of methods used in this paper urges the reader to interpret the results, even within the scope of the study design, with sufficient caution.

£See Table S2.

** The ‘B’ statement expresses whether the study supported the existence of exogenous boosting (‘+) or not (‘−’).

**Table 2:** Description of selected mathematical modeling studies.

Reference	Data and methods	Main Results	Quality*	Study Design£	B**
Garnett & Grenfell (1992)^29^	Simplified model: 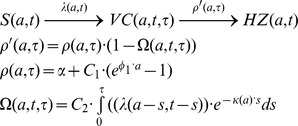 with 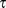 time since initial infection, VC the virus carrier compartment and Ω the exogenous boosting function & PDE describing the transition between compartmentsTransmission: 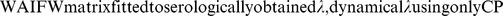 Fitting data: weekly rates of CP and HZ incidence collected from the records of 106 GP in England & Wales 1976–1987, age groups: 0–4y, 5–14y, 15–44y, 45–64y and >65y,Analysis: simulation of observed CP and HZ data under assumption of exogenous boosting, cross-correlation and multivariate spectral analysis to compare CP to HZ incidence	CP but not HZ had cyclical incidence data without immediately apparent correlations between the dynamics of the twoA shift in the mean age of CP incidence in children (increase of CP incidence in <5y) and a significant increase in CP incidence in 15–44y coincided with a slight, but significant decrease in the overall incidence of HZ, but mainly in 15–44y; this observation was simulated by changing the CP incidences which had a qualitative effect on HZ incidence similar to the observed dataWhether CP had no effect, an increasing effect or a decreasing effect on HZ did not qualitatively change age-dependent simulated HZ incidences	M	C	+/−
Brisson et al (2000)^30^	Simplified model: 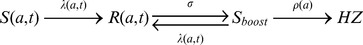 with 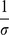 the duration of boosting, assumed to be 2 or 20 years (unclear)66 age cohorts parameterized in 8 groups; realistic age structured model (RAS) uses ODE to describe the transitionTransmission: 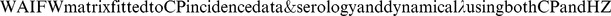 Fitting data: Manitoba (Canada) billings database 1980–1997 for fitting 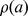 to HZ incidenceAnalysis: pre-CP-vaccination HZ incidence for Canada after simulation was compared with published incidence data for Canada & England	The exogenous boosting model predicts a similar HZ incidence (3.18/1000PY) to published data from Canada (3.21/1000PY) and England (3.43/1000PY) with also a similar age distribution	M	C	+/−
Brisson et al (2002)^32^	Simplified model: similar to previous Brisson et al^30^ 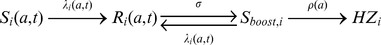 but different compartments indexed by ‘i’ for those living with children and for those who aren’t; the rates describing the movement between the different demographic compartments are modeled by demographic UK data and the GP surveyTransmission: no contact matrix and no dynamical 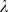 Fitting data: GP survey including over 500000 patients in England & Wales, 1991–1992; 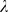 is estimated by fitting model CP and HZ incidence data to those observed by the survey data with 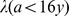 , assumed to have a Gamma distribution, and 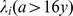 depending on exposure compartment but not on age; at the same time 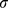 and 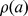 with 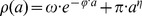 were estimated as wellAnalysis: Full-model log likelihood to estimate 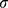 (and thus by definition the duration of boosting)	The best fitting model estimated 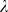 for adults with and without children in the household to be 0.15 and 0.07 per year, respectivelyExposure to CP is estimated to boost protection from HZ for an average of 20 years (95% CI, 7–41 years) with a qualitatively good fit to the data	M	C	+
Bonmarin et al (2008)^31^	Simplified model: similar to previous Brisson et al^30 ^ 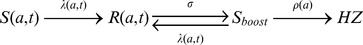 with 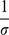 assumed to be 20y, but with 85 1y age groups and different demographic parametersTransmission: WAIFW matrix fitted to CP incidence data, dynamical 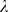 using both CP and HZFitting data: possibly based on French GP and specialist data, but not transparently reported: 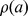 is modified from Brisson et al^30^Analysis: pre-CP-vaccination HZ incidence for France after simulation was compared with published incidence data for Canada & England	The HZ simulated incidence seemed to be higher than the observed data, however the authors noted that the latter could have been lower than in reality due to a registration run-in period	L	C	+
Brisson et al (2010)^32^	Simplified model: like Brisson et al^30^ 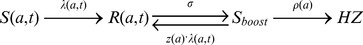 but with addition of vaccination for S (thereby creating a parallel circuit, not shown); exogenous boosting parameter z(a) depends on age group and was set to the probability of being boosted by the estimated age-specific zoster vaccine efficacy^33^_;_ RAS-ODE with 101 age cohortsTransmission: empirical social contact matrix from overall European mixing patterns for non-physical, HH and non-HH physical contacts were fitted to Canadian CP incidence+seroprevalence data, 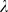 depends on CP and HZFitting data: first 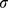 was estimated by imputing the new z(a) within the Brisson et al^13^ modeling and data framework for England and Wales, next holding 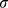 fixed the reactivation rate 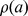 with 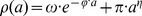 was refitted to the HZ incidence from the Canadian population (data from Brisson et al^30^)Analysis: estimation of 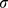 and qualitative comparison of post-CP-vaccination simulated incidence data to those published for USA (Antelope Valley, Philadelphia and Washington State)	Estimated 24.4 years protection against HZ by exogenous boostingVaccination model only partially agrees with USA post-vaccination data (particularly not a good fit for Massachusetts)	M	C	+
Van Hoek et al (2011)^34^	Simplified model: like Brisson et al^30^ 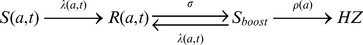 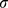 was predefined to be 1/20; RAS-ODE with bootstrapping for sensitivity analysisTransmission: England empirical social contact matrix further fitted to seroprevalence data for ages up to 20y and to varicella incidence thereafter (contact to transmission factor depends on age), 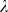 depends on CP and HZFitting data: England, same database as Brisson et al^13^ was used to estimate 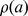 with 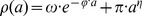 Analysis: comparison between observed and predicted pre-CP-vaccination HZ incidence	HZ predicted incidence was visually in accordance to observed data	M	C	+
Karhunen et al (2010)^35^	Simplified model: 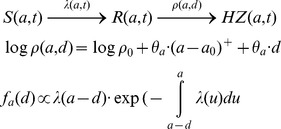 with the reactivation rate 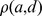 as a function of age (parameter 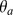 ) and time since last exposure ‘d’ (parameter 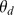 ); the distribution of d is modeled as a function of 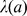 Transmission: Bayesian modeling (in contrast to maximum likelihood in previous models) approach to estimate contact matrix and age-specific proportionality factor simultaneously by fitting to seroprevalence data, 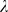 depends on CP and HZFitting data: Finland outpatient HZ incidence (several centers between 2000–2006), 17 5-year age groups, 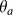 and 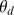 were estimated by applying a Metropolis–Hastings algorithm for the analysis of their posterior distribution modeled with HZ occurrence as a Poisson processAnalysis: boosting parameter was estimated for 2 scenario's depending on threshold for waning immunity with age	Having waning of immunity by age start at 45y increases HZ risk by 4.4% each year and by 3.3% each year since previous exposure; when threshold is set at 65y, ageing increased risk with 0% per year and 8.4% per year since exposure; model selection criteria found the ageing threshold ideally set at 45yThe effects of ageing and boosting were in both scenarios strongly dependent.	M	C	+

*S* susceptibles compartment; 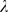 force of infection; 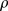 reactivation rate; *HZ* herpes zoster; *PDE* partial differential equations; *WAIFW* who-acquires-infection-from-whom; *CP* chickenpox; *R* CP recovered compartment; *S_boost_* susceptible to boosting compartment; *ODE* ordinary differential equations.

* H = High: the quality of methods used in this paper permits the results, within the scope of the study design, to be interpreted with at the most a few remarks. M = Medium: the quality of methods used in this paper permits the results, within the scope of the study design, to be interpreted, but with some caution. L = Low: the quality of methods used in this paper urges the reader to interpret the results, even within the scope of the study design, with sufficient caution.

£See Table S2.

** The ‘B’ statement expresses whether the study supported the existence of exogenous boosting (‘+) or not (‘−’).

**Table 3:** Description of selected epidemiological risk factor studies.

Reference	Data and methods	Main Results	Quality*	Study Design£	B**
Solomon et al (1998)^36^	Type: Retrospective surveyCoordinates: USA, 1994–1995Data: 1109 pediatricians, 1984 dermatologists & 462 psychiatristsExposure: VZV exposureAnalysis: chi-square	Pediatricians had more annual VZV contacts than dermatologists and both groups had more VZV contacts than psychiatristsHZ cumulative incidence differed significantly between the three groups: pediatricians (5.95%), dermatologists (9.27%) and dermatologists (10.82%)	L	B	+
Thomas et al (2002)^37^	Type: Prospective Case-controlCoordinates: England, 1997–1998Data: 244 HZ patients, 485 matched controlsExposure: Contact with CP or HZ past 10y & social/occupational contact with children as proxy for exposureAnalysis: univariate & multiple logistic regression	Univariate analysis: protective effect of exposure to CP or HZ, children in HH, child-care work; occupational exposure to many ill children (not necessarily CP), greater numbers of social contacts with specific children not living in HH or children in groups; no significant effect on HZ of duration of occupational exposure to many healthy childrenMultivariate analysis: OR remained significant for increasing CP contacts (>3 OR 0.26), contacts with large groups of children (OR 0.19) & occupational contact with many ill children	H	B	+
Brisson et al (2002)^13^	Type: Retrospective surveyCoordinates: England & Wales, 1991–1992Data: >500000 random individualsExposure: living with children <16y or notAnalysis: incidence ratio	Living with children was significantly protective against HZ (p<0.001) with an incidence ratio of 0.75 (95% CI 0.63–0.89)	H	C	+
Chaves et al (2007)^38^	Type: Retrospective surveyCoordinates: USA, 2004Data: 3435, ≥65y, individuals through national random-digit telephone surveyExposure: Close contact with CP in past 10yAnalysis: Wald chi-square tests	Exposure to CP had a RR of 0.63 (p>0.05)	M	B	+/−
Donahue et al (2010)^39^	Type: Retrospective case-controlCoordinates: Wisconsin, USA, 2000–2005Data: 40–79y, 633 HZ & 655 controls, telephone survey after medical database analysis of track recordsExposure: number of CP or HZ contacts+setting of contact, contact with children in HH, with non-HH family and in social settings in past 10yAnalysis: univariate and multiple logistic regression with backward model reduction	None of the exposure variables were significant	M	B	–
Wu et al (2010)^40^	Type: Retrospective database analysisCoordinates: Taiwan, 2000–2005Data: 695188 randomly selected individuals ≥20y from mandatory universal health insurance program on ambulatory care and inpatient discharge recordsExposure: 3 groups: dermatologists & pediatricians, other medical professionals, and general adults who are not health-care workersAnalysis: Univariate Chi-square, multiple logistic regression, Cox regression	Time trend for HZ incidence went from 4.9/1000PY in 2000 to 7/1000PY in 2005Univariate testing showed higher incidence in dermatologists/pediatricians aged 20–39 compared to general adults with reversed results for the older age groups (RR 0.23 for dermatologists/pediatricians ≥40y); no overall difference was noted between these groups when using multiple regression for analysisMultiple logistic regression and Cox regression show significantly higher OR (1.39 & 1.38 respectively) for other medical professionals compared to general adults	M	C	–
Salleras et al (2011)^41^	Type: Prospective case-controlCoordinates: Barcelona, Spain, 2007–2008Data: dermatology department, 153 HZ & 604 matched controls, ≥25yExposure: contact with children (<15y) in past 10yAnalysis: Chi-square and conditional logistic regression for controlling confounding factors using backward selection	Some contact with children gave adjusted OR 0.57 with dose response: exposure to children up to a maximum of 4000 hours had an OR of 0.60 and >4000 hours had an OR of 0.48	M	C	+
Gaillat et al (2011)^42^	Type: Retrospective surveyCoordinates: France, 2008–2009Data: HZ incidence data from 920 members of contemplative monastic orders (CMO), 1533 representatives of French general population (FGP)Exposure: normal contact with children (FGP) vs. low contact with children (CMO)Analysis: univariate chi-square & multivariate logistic regression	Cumulative HZ incidence was 16.2% in CMO and 15.1% in FGP (NS OR 1.14 adjusted for sex and age)Mean age when having HZ was 54.8y in CMO and 48.6y in FGP; members of CMO with a history of HZ reported having more diseases at the time of onset of zoster than did persons from the FGP (28.8% vs. 16.8%, p<0.007)	M	C	–
Lasserre et al (2012)^45^	Type: Prospective case-controlCoordinates: France, 2009–2010Data: 250 HZ patients, 500 controls age and gender matched, aged ≥50yExposure: number of children in close contact in the past 10y including both children living in the HH and children not living in the HH and occupational contactAnalysis: conditional logistic regression (including interactions) with backward elimination of non-significant interactions and covariates to identify significant risk factors; univariate risk factors associated with HZ were included for multivariate analyses when p≤0.25	Univariate analysis: having lived with children under 12y over the last 10y had OR of 0.51 (p = 0.04)After multivariate analysis the OR had a 95% CI of 0.18–1.27	H	C	+/−

*VZV* varicella-zoster virus; *HZ* herpes zoster; *CP* chickenpox; *HH* household; *OR* odds ratio; *aOR* adjusted odds ratio; *NS* not significant; *PY* person-years.

* H = High: the quality of methods used in this paper permits the results, within the scope of the study design, to be interpreted with at the most a few remarks. M = Medium: the quality of methods used in this paper permits the results, within the scope of the study design, to be interpreted, but with some caution. L = Low: the quality of methods used in this paper urges the reader to interpret the results, even within the scope of the study design, with sufficient caution.

£See Table S2.

** The ‘B’ statement expresses whether the study supported the existence of exogenous boosting (‘+) or not (‘−’).

**Table 4:** Description of selected prospective longitudinal studies on VZV-immunity post exposure and other selected studies.

Reference	Data and methods	Main Results	Quality*	Study Design£	B**
**Prospective longitudinal studies on VZV-immunity post exposure**					
Arvin et al (1983)^46^	Population: 25 healthy women RE to CP by their children & 42 COTime points: t1 within 4d after exposure and t2 between 3w and 4w after onset exanthemaLab: VZV-specific IgG, IgA, IgM via solid-phase RIA; fresh PBMC in vitro lymphocyte transformation to VZV antigen, TT & PHA; also in vitro IFN-gamma in supernatantsAnalysis: t-test and chi-squared	9/25 RE showed no kinetics in IgG, also no difference with 42 CO was noted; 9/25 RE had >4x IgG increase, 7/25 RE had high initial IgG followed by >4x IgG decrease to a baseline IgG value not different from those from CO3/25 RE ≥1 time point with positive IgM whereas 0/15 CO had positive IgM16/23 RE developed IgA at t1 and 12/14 RE still had IgA at t2. 2 RE only had IgA at t2, and 3/23 CO had IgA (significantly lower)14/23 RE had increases in VZV T-lymphocyte proliferation with a significant mean absolute increase from 4038 to 10861 cpm (in contrast to 12 CO with cpm from 6256 to 8559); only 1 RE showed a decrease in cellular immunity; for 15 RE TT remained stable at t1 and t2No effect of exposure on IFN-gamma in supernatants was noted; 15/21 RE showed 'concordance' between AB & cellular immunity	L	A	
Gershon et al (1990)^47^	Population: 38 household RE parentsTime points: 26 with single time point (between 1d-40d after re-exposure), 12 with paired samplesLab: VZV Total and IgM FAMA titersAnalysis: descriptive	7/38 RE had high titer (≥1:64)Paired sera were available for 12 RE: 1 went from 1:8 to 1:32 (3d-14d), 1 from 1:2 to 1:64 (14d-40d), 10/12 RE had no increase (although for 2 parents the titers ≥1:64) 6/38 RE had IgM (of whom 2 also had total > = 1:64)Authors concluded that 12/38 showed boosting	L	D	+
Vossen et al (2004)^48^	Population: 16 parents & 1 grandparent with HH RE to CP, 10 COTime points: for RE within a one year time frame several (minimal 3) sampling points, first within 3 weeks after onset exanthema, single point for COLab: VZV IgG immunofluorescence; cryopreserved PBMC for ELISPOT and FCM/ICS using VZV cell lysate co-stimulation with CD28 & CD49d; markers: CD4, CD8, CD16, CD27, CD45RA, CD56, CD69, TNF-alpha, IFN-gamma, IL-2; VZV PCR on serum samplesAnalysis: Linear regression, Student’s T-test	8/16 RE showed at least 1 unit increase or decrease in VZV IgGA 8 times higher % of VZV-specific CD4+ cells was noted during the early phase in boosted RE compared to CO11/16 RE had peak level within 4 weeks followed by steep contraction up to week 6 and slow decrease thereafter; 1y later VZV-specific CD4+ cells were detectable in all these RE at 0.08% (qualitatively constant from +/−15 weeks); TT-specific CD4+ cells remained constant at all times5/16 RE had no VZV-specific IFN-gamma increaseRE cells were mainly CD45RA- and showed a strong correlation between peak-level VZV-CD4+ percentages and CD27-negativity; this correlation returned to baseline at 1yVZV-specific IFN-gamma, TNF-alpha and IL-2 had similar kinetics in all RE; CD8+ and NK cell kinetics were similar to CD4+ kinetics in RE; VZV was not detected in plasma from REThere was no overall correlation between peak IgG and VZV-CD4+%, but a correlation was more common in those with boosted cellular immunity	M	A	+
Ogunjimi et al (2011)^49^	Population: 18 RE, 15 young CO (YCO), 20 older CO (OCO)Time points: for RE fixed at <1w, 1mo, 7mo, 12mo since onset exanthema; single point for COLab: VZV IgM & IgG using indirect chemiluminescence (antigens from cell lysate), cryopreserved PBMC for IFN-gamma ELISPOT with VZV cell lysate, analysis per sample-momentAnalysis: t-test (or Wilcoxon when non-normality), linear mixed models	IgG showed no longitudinal profile in RE but was at 1mo higher in RE compared to YCO (mean GMR 1279 vs. 902 mIU/ml with p<0.05) and at all time points compared to OCO (GMR 695 mIU/ml), IgG was not significantly different between YCO and OCORE IFN-gamma ELISPOT showed a decrease at 1mo compared to 1w and had a tendency (p<0.1) to be higher at 1y compared to 1w (factor 1.6–1.8)A lower ELISPOT response was noted for RE at 1mo (GMR 18/250000 cells) than for YCO (47/250000 cells) and the ELISPOT response for OCO (23/250000 cells) was lower than those in RE at 12mo (63/250000 cells) and YCOA negative correlation between IgG and ELISPOT was noted for RE	M	A	+
**Other studies**					
Gershon et al (1982)^52^	Population: USA, 1982Data: 6 RE (known HH exposure to VZV), 49 controlsLab: IgM FAMAAnalysis: descriptive	4/6 RE had IgM+ compared to 11/49 controls	M	D	+
Terada et al (1993)^53^	Population: JapanData: 8 immune healthy adults, 10 pediatriciansLab: responder cell frequency with limiting dilution against VZV antigen & VZV IgM and IgG ELISAAnalysis: T-test	RCF values in pediatricians were higher than in healthy adults (p<0.001)No differences in IgG or IgM were reported and no correlation between IgG and RCF was reported	M	C	+
Terada et al (2000)^54^	Population: JapanData: children with recent CP, children with CP at least 2 years earlier, children previously vaccinated against CP, immunocompromised children, individuals who had HZ in past year, healthy elderly (>60y) and doctors/nurses with frequent exposure to VZVLab: salivary VZV IgA using ELISA and neutralizationAnalysis: Student’s T-test	Medical workers with VZV exposure had the highest salivary IgA of everyone, even comparable to the values from the HZ group and higher than those in elderly (>60y)	L	D	+
Yavuz et al (2005)^55^	Population: Turkey, 2005Data: 73 HCW vs. 65 office workers, all femaleLab: VZV, measles and HBV IgG ELISAAnalysis: Chi-square, Fisher’s exact, Student’s t-test, Mann-Whitney U test	Comparable seropositivity for VZV and history for CP and measles between groups, but a higher titer was noted for measles (most pronounced) and for CP	L	D	+
Saadatian-Elahi (2007)^56^	Population: France, 2005Data: 480 pregnant women with questionnaireLab: ELISA VZV IgGAnalysis: analysis of variance	No statistically significant differences were observed between IgG levels and number of children in household	L	D	-
Valdarchi et al (2008)^57^	Population: Italy, 2005Data: CP outbreak in women prisonLab: VZV IgM & IgG ELISAAnalysis: anecdotal	5 CP cases amongst 314 inmates10 asymptomatic women were IgM+: 7 were IgG+, 7 had a history of CP and 2 had known a contact with CP during the outbreak	L	D	+
Toyama et al (2009)^58^	Type: Retrospective database analysisCoordinates: Miyazaki (Japan), 1997–2006Data: CP surveillance data; HZ consultations recorded by dermatologistsCP vaccination: estimated coverage of 20–30% in children born during study (non-public funding)Analysis: qualitative & visual comparison of CP and HZ incidence data over a short period.	The authors state (data not shown) that CP epidemiology from Japan did not change between 1999–2008HZ incidence increased during the study period in females older than 60yThe authors showed visually that after averaging over the study period HZ incidence in Miyazaki mirrored the national CP incidence at a seasonal level with a HZ peak in the summer	L	A	+

*RE* re-exposed; *CP* chickenpox; *CO* controls; *VZV* varicella-zoster virus; *RIA* radioimmunoassay; *PBMC* peripheral blood mononuclear cells; *TT* tetanus toxine; *PHA* phytohemagglutin; *IFN* interferon; *Cpm* counts per minute; *FAMA* fluorescent antibody to membrane antigen; *FCM* flow cytometry; *ICS* intracellular cytokine staining; *ELISPOT* enzyme-linked immunosorbent spot; *GMR* geometric mean response; *ELISA* enzyme-linked immunosorbent assay; *RCF* responder cell frequency; *HCW* health-care workers.

* H = High: the quality of methods used in this paper permits the results, within the scope of the study design, to be interpreted with at the most a few remarks. M = Medium: the quality of methods used in this paper permits the results, within the scope of the study design, to be interpreted, but with some caution. L = Low: the quality of methods used in this paper urges the reader to interpret the results, even within the scope of the study design, with sufficient caution.

£See Table S2.

** The ‘B’ statement expresses whether the study supported the existence of exogenous boosting (‘+) or not (‘−’).

Due to large heterogeneities between different study designs, we chose to apply a two-stage grading system, based on pre-set criteria agreed upon between all co-authors. This way, the relevance of the study design to assess exogenous boosting (study design levels from D to A+, see Table S2) and the overall quality based on potential biases, given its specific study design (Low-Medium-High, see Table S3), were assessed separately.

## Results

### HZ Incidence in Countries with Widespread CP Vaccination Studies (see Table 1)

Mullooly et al (quality: medium, relevance: A) found a difference when comparing pre-CP-vaccination HZ incidence in one USA region with the post-CP vaccination HZ incidence in another USA region [14]. HZ incidence was also noted to increase during the post-CP-vaccination years, but only in 10–17 year olds. Poisson multiple regression showed the observed HZ increase to be mediated mainly by an increase in oral steroid exposure. Using telephone survey data, Yih et al (quality: medium, relevance: A) observed that the age-standardized HZ incidence in Massachusetts (USA) increased by 90% in the 5 years after vaccination reduced CP incidence [15]. Unfortunately, Yih et al did not control for other potential causes for such an increase. These may include differences in reporting practices over time. For instance, gradually increased reporting of clinical diseases may occur during long term run-in periods following fundamental changes in surveillance, such as the implementation of electronic data collection/reporting. Furthermore, a temporal rise in HZ incidence could also be due to rising numbers of immunocompromised individuals. Jumaan et al (quality: medium, relevance: A) examined the medical records of a health maintenance organization (HMO) in Washington (USA). They observed no change in overall HZ incidence in the post-vaccination era, but noted an increase in HZ in unvaccinated children [16]. Importantly, the overall CP incidence only started to decrease during the last four years of observations. Patel et al (quality: low, relevance: A) showed a marked increase in HZ hospitalizations in >65 year olds after the introduction of CP vaccination in the USA [17]. Rimland et al (quality: medium, relevance: A) observed an HZ increase in veterans aged older than 40 years during 8 years after the start of widespread CP vaccination [18]. Carville et al (quality: medium, relevance: A) reported an increase in after hours telephone HZ diagnosis after, but also prior to, introduction of CP vaccination in Victoria (Australia) [19]. Updates by Grant et al [20] and Carville et al [21] further supported this observation. Nelson et al (quality: low, relevance: A) found an increase in the proportion of GP consultations for HZ in Australia [22]. However, due to data limitations, they could not exclude a trend that may have started in the pre-vaccination era. Also, as observed by Heywood et al [23], the lack of age-standardization prohibited a correct interpretation of Nelson et al’s [22] observations. Jardine et al (quality: low, relevance: A) noted an increase in emergency room visits for HZ and an increase in antiviral use in Australia after introduction of CP vaccination [24]. Five years after universal CP vaccination was funded in Ontario (Canada), Tanuseputro et al (quality: medium, relevance: A) found no overall increase in HZ incidence, but observed an increase over the last year for those aged over 60 years [25]. Based on a medical insurance database Leung et al (quality: high, relevance: A) reported a gradual convergence of HZ incidence between adults, aged 20–50 years, with and without dependent children that started after the introduction of universal CP vaccination in the USA [26]. This convergence became complete in 2005, strongly suggesting that the association between living with children and a lower likelihood of HZ weakened due to the increasing uptake of childhood CP vaccination. However, they also found an overall increase in HZ incidence with time that started before the introduction of universal CP vaccination. A HZ incidence increase was also observed in immunocompetent individuals separately (factor 1.3–1.4 increase in age-standardized rate over 8 post-CP-vaccination years) and thus indicates that the HZ increase was not solely caused by an increase in immunosuppressed individuals. It was noted that the overall HZ incidence did not differ between US states with higher and lower CP vaccine uptake compared to the national median coverage, but no information was given in regard to the actual differences in CP incidence. Chao et al (quality: high, relevance: A) reported an increase in HZ coinciding with a decrease in CP after introduction of CP vaccination in Taiwan [27].

Overall, 8 studies supported the exogenous boosting hypothesis, whereas 3 studies did not and 2 remained inconclusive in regard to the exogenous boosting hypothesis.

### Mathematical Modeling Studies (see Table 2)

All mathematical models described in this section are based on a deterministic modeling approach in which individuals are grouped in a number of compartments describing the state of the individual in relation to VZV infection. This is a population-based approach in which individual characteristics are averaged within the compartments and age groups. The transition between compartments is mainly modeled by differential equations governed by rates describing the movement from one compartment to another. In the described models, transition rates are given by 1/(average time an individual stays in a compartment).

At first, individuals are assumed to be susceptible ‘S’ to infection (mostly after a period of protection through maternal antibodies). Next, individuals can be infected with VZV at a certain age and they will move from S to the CP recovered ‘R’ compartment. The force of infection 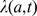 describes the annual risk for a susceptible person of age a to be infected at time t with VZV. For example 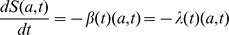 means that susceptibles leave their compartment at a rate proportional to the number of susceptibles. The force of infection 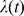 is the product of the contact rate 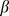 and the number of infectious individuals at time t, I(t). Note that 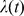 also represents the number of re-exposure episodes per year. The contact rates are described by the Who-Acquires-Infection-From-Whom ‘WAIFW’ matrices. Initially these matrices were estimated by using simplified and a priori assumptions on the way age groups interact with each other whereas in later work empirical social contact matrices were used [28]. Finally, modulated by the modeling approach, a reactivation rate 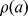 describes the transition from R to HZ, also allowing a route for exogenous boosting.

Garnett and Grenfell (quality: medium, relevance: C) were the first to incorporate exogenous boosting in a mathematical model. In their model, they added a ‘time since initial infection’ variable in addition to the ageing variable [29]. The reactivation rate was assumed to be a function of age. They formulated an exogenous boosting function that was applied to proportionally reduce the reactivation rate. The boosting function was constructed as an integration of all past exposures to CP but damped with time since exposure thereby assuming the most recent re-exposure to have the biggest effect. Also, the effect of exogenous boosting was assumed to decrease with age. An epidemiological observation revealed a downward shift in the mean age of CP coinciding with an increase in CP in 15–44 year olds and a small but significant increase in HZ in individuals aged 15–44 years. A simulation with an artificial increase in CP in 15–44 year olds predicted a decrease in HZ, which was qualitatively in accordance with the observations. Time series analysis showed CP incidence to be cyclical and HZ incidence not, and no correlation was found between the two on a weekly scale.

Brisson et al (quality: medium, relevance: C) modeled the transition from CP to HZ in a three part compartmental model where individuals went from R to HZ via a transitory compartment named ‘susceptible to boosting ‘S_boost_’’ [30]. Thus, instead of one rate describing the reactivation process, two rates were assumed to exist. The rate 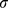 from R to S_boost_ was imputed independently of age and represents continuous waning of immunity with time since primary infection. Next, an age dependent ‘reactivation’ rate, estimated by fitting to HZ incidence data, defined the transition from S_boost_ to HZ. Exogenous boosting was assumed to move individuals from S_boost_ back to R by a rate, which was assumed to be proportional to the force of infection. This way re-exposure was assumed to have only effect when immunity was sufficiently low. Without specifying whether the average time in R was 2 or 20 years the authors noted the predicted HZ incidence to be qualitatively similar to the observed HZ incidence data. There was no assessment made whether the inclusion of exogenous boosting created a better fit to the data. In a later paper Brisson et al (quality: medium, relevance: C) estimated 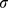 and the reactivation rate by fitting them simultaneously to HZ incidence data using sub-compartments to distinguish people living with and without children [13]. Brisson et al thus estimated that re-exposure to CP would boost immunity to HZ for an average of 20 years with a 95% CI of 7–41 years and they considered the fit to the data to be good. Several partially modulated papers using the Brisson et al methodology were published in the past ten years. Bonmarin et al (quality: low, relevance: C) adjusted the Brisson et al [30] methodology to French data under assumption of 20 years duration of boosting, however with limited details, and they qualitatively noted a good agreement between simulated and observed data [31]. Brisson et al (quality: medium, relevance: C) [32] applied the European empirical social contact matrices on Canadian data and allowed an age-dependent effect of boosting which was inspired by age-specific HZ vaccine efficacy results of the Shingles Prevention Studies [33]. They remodeled the England & Wales dataset from reference [13] and re-estimated 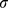 to be 1/(24.4) years. Next, they imputed these values for Canada, thereby estimating the reactivation rate. They observed that a simulation of the USA early post-CP-vaccination years only partially (qualitatively) agreed with the surveillance data on HZ incidence. Additional analyses showed important sensitivities to model components such as the contact matrix and vaccine efficacy estimates. Van Hoek et al (quality: medium, relevance: C) applied the empirical social contact data for England [34]. By using a 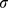 of 20 years they found a good fit between the predicted and observed HZ incidence data.

Karhunen et al (quality: medium, relevance: C) presented another approach to modeling exogenous boosting [35]. They constructed an innovative Bayesian method to estimate the reactivation rate, which they formulated as a log-linear function of age plus time since last exposure. Karhunen et al thus explicitly assumed exogenous boosting to be on the same level as ageing. Also, they assumed that the ageing effect could be delayed until a certain age. Fitting to HZ incidence data and allowing waning by age to start at 45 years, they estimated that each year since last exposure would increase the HZ risk with 3.3%. When delaying the ageing until 65 years, the HZ risk increased 8.4% with each year since last exposure. Model selection criteria preferred the model with an ageing threshold of 45 years. By having waning start after an age threshold, Karhunen et al implicitly, and possibly erroneously, assumed that irrespective of time since initial infection the reactivation rate at ages before the threshold was only determined by the time since last exposure. However, observed HZ incidence was quite constant up to 40–45 years, but the time since last exposure was not.

Overall, 5 studies supported the exogenous boosting hypothesis, whereas 2 studies remained inconclusive in regard to the exogenous boosting hypothesis.

### Epidemiological Risk Factor Studies (see Table 3)

Solomon et al (quality: low, relevance: B) found, in a study with a low response rate, the cumulative HZ incidence in psychiatrists to be almost twice of that of pediatricians and comparable to that of dermatologists [36]. A case-control study by Thomas et al (quality: high, relevance: B) found that an increasing number of exposures to CP, social contacts with children or occupational contacts with ill children (not necessarily CP) reduced the risk of HZ [37]. Their multivariate analysis showed the OR for HZ to decrease from 0.9 to 0.29 when 1 to ≥5 known contacts with CP patients occurred over the last 10 years. Examining a national survey, Brisson et al (quality: high, relevance: C) showed adults currently living with children in the household to have a lower HZ incidence than same-aged adults who do not live with children (incidence ratio of 0.75) [13]. Through telephone surveys after the introduction of universal CP vaccination in the USA, Chaves et al (quality: medium, relevance: B) [38] and Donahue et al (quality: medium, relevance: B) [39] found that exposure to children with CP during the previous 10 years was not protective against HZ. However, the low incidence of CP could have underpowered the study by Chaves et al who found a RR of 0.63 with p value >0.05. Also, both studies were possibly biased by a lower boosting potential of breakthrough CP, which could explain the high HZ incidence (19/1000PY when > = 65 years) found by Chaves et al. Wu et al (quality: medium, relevance: C) studied the HZ incidence in a mandatory universal health insurance program and made a risk factor analysis for dermatologists & pediatricians, other medical professionals, and the general population [40]. Multiple logistic regression showed a significantly higher HZ OR of 1.39 for the ‘other medical professionals’ compared to the general population. Based on few cases, they found a much higher incidence in dermatologists and pediatricians aged 20–39 years compared to the general population and the reverse was true for 40–59 year olds. The authors suggested the former result to be caused by stress. A case-control study by Salleras et al (quality: high, relevance: C) showed that having more than 4000 contact hours with children (within and outside the household) in the last 10 years lowered the HZ OR to 0.48 [41]. Gaillat et al (quality: medium, relevance: C) performed an innovative study in which HZ incidence was compared between members of monastic orders, who were selected to have had fewer exposures to CP, and the general population [42]. They found no difference in HZ incidence between the two groups. However several methodological issues, such as gender bias, limit the interpretation of this result. These issues have been discussed elsewhere [43], [44]. Finally, after multivariate analysis in a prospective case-control design, Lasserre et al (quality: high, relevance: C) found living together with children not to be protective against HZ [45].

Overall, 4 studies supported the exogenous boosting hypothesis, whereas 3 studies did not and 2 remained inconclusive in regard to the exogenous boosting hypothesis.

### Prospective Longitudinal Studies on VZV-immunity Post-exposure (see Table 4)

Arvin et al (quality: low, relevance: A) compared VZV-specific lymphocyte proliferation data and antibody titers at less than 4 days and at 3 to 4 weeks since re-exposure to CP in women [46]. An increase in cellular immunity was seen in 61% and an IgG response in 64% of re-exposed. Interestingly, the IgG response was influenced by the initial value with low initial values increasing and high initial values decreasing after re-exposure. This could be due to either a faster immune response or a longer time period between re-exposure and the first sample (causing a peak response on the first time point, followed by a decrease at the second time point). VZV-specific serum IgA positivity was frequently and IgM positivity was rarely noted after re-exposure. Gershon et al (quality: low, relevance: D) observed in a similar study design, solely focusing on VZV-specific antibodies, that 32% of re-exposed parents were immunologically boosted within 40 days after re-exposure [47].

Vossen et al (quality: medium, relevance: A) emphasized that boosting of immunity was not seen in all re-exposed (11/16 showed an increase in cellular immunity) and that some individuals had an increase in antibody titers whereas others had a decrease [48]. Intracellular cytokine staining showed qualitatively an initial up-rise in VZV-specific IFN-gamma CD4+ cells (with similar kinetics for CD8+ and NK cells) at 4–6 weeks since re-exposure, followed by a decrease until stabilization (probably still higher than compared to a control group) around 15 weeks. Half of the re-exposed showed either an increase or a decrease in antibody titer. No VZV DNA was detected in the blood.

Ogunjimi et al (quality: medium, relevance: A) found a factor 1.6 VZV-specific IFN-gamma ELISPOT increase from 1 week to 1 year post-exposure, but could not demonstrate a higher cellular immunity in exposed versus control individuals (even the reverse was true at 1 month after re-exposure) [49]. The latter could be explained by cryopreservation, leading to cell apoptosis and reduced immune responses [50], and inter-assay biases [51]. They also observed no formal significant longitudinal effect by re-exposure on antibody titers, although there was a tendency for overall higher antibody titers in the re-exposed group as compared to the control group.

Overall, all 4 studies supported the exogenous boosting hypothesis. However, only 2 studies had data up to one year post re-exposure.

### Other Studies (see Table 4)

Gershon et al (quality: medium, relevance: D) found VZV-specific IgM antibodies in 4 out of 6 household re-exposures in comparison to 11 out of 49 control individuals [52]. Terada et al (quality: medium, relevance: C) noted that pediatricians had a higher VZV-specific responder cell frequency compared to non-matched healthy adults thereby suggesting a cellular effect of boosting [53]. In a later study, Terada et al (quality: low, relevance: D) also showed that health care workers frequently exposed to VZV had increased serum IgA levels [54]. A sero-epidemiological study by Yavuz et al (quality: low, relevance: D) described higher VZV-specific IgG in health-care workers compared to office workers [55]. Saadatian-Elahi et al (quality: low, relevance: D) studied pregnant women and found no effect of the number of children in the household on VZV IgG titers [56]. Valdarchi et al (quality: low, relevance: D) noted during a prison CP outbreak study that some asymptomatic inmates developed VZV IgM, suggesting re-exposure, although only two had a known CP contact [57]. Toyama et al (quality: low, relevance: A) suggested a short term influence of CP on HZ incidence through visual inspection of plots, showing an increase in HZ during the summer period when CP circulation is at its lowest [58]. However, if this observation would prove to be based on a statistically significant association, it could easily be explained by other causal factors than CP (for e.g. solar exposure). Furthermore, a recent review did not find sufficient epidemiological support for the seasonality of HZ incidence [6].

Overall, 6 studies supported the exogenous boosting hypothesis, whereas 1 study did not.

## Discussion

We presented an in-depth systematic review of the literature on exogenous boosting for VZV.

The majority (27/40) of HZ incidence studies post widespread CP vaccination showed the existence of exogenous boosting. However, the clear extent of this effect is not easy to interpret due to a substantial population and environmental heterogeneity (e.g. CP vaccination coverage, variations in surveillance practices) and the lack of pre- and post-CP vaccination data for a sufficiently long time. Indeed, in all of the studies in countries with a universal CP vaccination program, the time period since the occurrence of large reductions in CP incidence is perhaps too short and the role of less infectious breakthrough infections might be too important to allow differentiating between the different boosting hypotheses. Also, some studies noted an increase in HZ incidence preceding initiation of CP vaccination. This could be explained by an ageing population, increasing immunosuppression (by disease and/or medication), but also by more accurate surveillance and the implementation of electronic data collection/reporting. However, as much is yet to be learned about risk factors for HZ in otherwise healthy individuals, it remains challenging to control for changing risk factors over time. With the exception of one study [16], these studies did not correct for the decreasing presence of naturally infected children in the denominators. So, although exogenous boosting seems to exist, it is not clear from these studies to which extent it affects HZ incidence in the community when CP incidence is reduced.

Prospective longitudinal immunological studies after re-exposure circumvent the difficulties associated with epidemiological studies. All such studies clearly showed exogenous boosting to exist, but clearly not for all re-exposure episodes. It is likely that the proportion of boosted individuals and the duration of boosting depend on several variables such as the intensity and duration of the contact [28], [59], age-specific characteristics [59], the viral load [60] and the pre-re-exposure immunity levels at the mucosa and in the blood circulation [3]. The mere existence of exogenous boosting is not surprising as it should be interpreted as a secondary immune response. We found only two studies that analyzed samples up to one year post-re-exposure [48], [49]. However, shortcomings in study design hampered estimating the duration of immunological boosting. Nonetheless, the combined immunological data suggest the effect of immunological boosting would not extend beyond two years. We underline however that both the threshold level, if it exists, and the cell type(s) of the immunological correlate of protection against HZ are yet to be defined with certainty. Also, endogenous boosting after asymptomatic reactivation could add to the complexity of the VZV immune response [61].

Weighting by study design and quality of epidemiological studies that assessed the effect of re-exposure to CP, both directly and indirectly, supported the existence of exogenous boosting although the magnitude and time frames for re-exposure varied between studies. It is important to note that some of these studies used contact with children as a proxy for VZV re-exposure. Overall, one can assume that women have more contacts with children than men. However, women tend to have higher HZ incidence rates than men. Possibly, just household re-exposures, which would be more equal between men and women, can be sufficient for exogenous boosting. Alternatively, gender susceptibility to HZ could have a greater effect on HZ occurrence than exogenous boosting.

The mathematical models that contributed to many countries’ hesitation to start universal CP vaccination, involved an important and long term effect of exogenous boosting on HZ incidence. However, none of the models allowed for an explicit comparison of the goodness-of-fit between scenarios with and without boosting. These modeling papers predicted HZ incidence ratios around 1.15–1.4 10 years after introduction of CP vaccination for a 20 year boosting scenario and still increasing modestly for some years thereafter before a decrease would occur. We note that the modeled HZ incidence ratios do not differ substantially between high vaccine uptake scenarios with short (e.g. 2 years) or long (e.g. 20 years) durations of boosting over time horizons up to 10 years. This implies that current observational HZ post-CP-vaccination data can not yet be used to estimate the duration of the effect of boosting. The predicted incidence ratios are however relatively low when compared to some of the present HZ incidence data (up to 10 years since introduction of CP vaccination). Possibly, the observed HZ data could be caused by a combination of lack of exogenous boosting and a background increase in HZ incidence registration (for e.g. due to gradually improving surveillance). The pre-CP-vaccination HZ increases found in some studies support the latter statement. If however the observed HZ incidence ratios are solely caused by the lack of boosting, they could be explained through a variety of underlying mechanisms. Indeed, the observed HZ incidence is a consequence of the balance between the physiological reactivation rate, which is a driving force towards HZ, and the duration of boosting, which defines the time period of protection against HZ. Thus, for HZ incidence to remain constant in a pre-CP-vaccination mathematical model, the reactivation rate should increase when the duration of boosting increases and vice versa. Thus a higher observed post-CP-vaccination HZ incidence could mean that the duration of boosting was higher than predicted by the models (the models would seem to be insufficiently predictive in this instance). Consequently, the number of years with a net increase in HZ incidence post-CP-vaccination would be higher as well. However, even a lower duration of boosting combined with a higher exogenous boosting frequency could lead to a higher initial HZ incidence, but within a shorter time frame as compared to the scenario with a longer duration of boosting. The effect of different contact rates on HZ incidence post-CP-vaccination was illustrated by Brisson et al [32].

Our review was limited by the exclusion of non-peer reviewed publications and of abstracts-only publications.

Our review has found sufficient support to conclude that the mechanism of exogenous boosting exists, although not for all persons, nor in all situations. Besides continuing post-CP-vaccination surveillance studies over time periods with expected higher HZ incidence, we reckon that the duration of boosting would still be the most important and the most challenging parameter to estimate correctly. Both improved mathematical models and post-re-exposure immunological studies, combined with a better identification of the immunologic correlates of protection through HZ vaccination trials, should allow a more accurate estimation of the duration of boosting.

## Supporting Information

Table S1 **Inclusion algorithm.**
(DOC)

Table S2 **Grading of different study designs.**
(DOC)

Table S3 **Potential biases identified for each included study.**
(DOC)

## References

[1] Chaves SS, Zhang J, Civen R, Watson BM, Carbajal T, et al (2008) Varicella disease among vaccinated persons: clinical and epidemiological characteristics, 1997–2005. J Infect Dis 197 Suppl 2S127–131. 18419385 10.1086/522150

[2] Chaves SS, Gargiullo P, Zhang JX, Civen R, Guris D, et al (2007) Loss of vaccine-induced immunity to varicella over time. N Engl J Med 356: 1121–1129. 17360990 10.1056/NEJMoa064040

[3] Levin MJ, Smith JG, Kaufhold RM, Barber D, Hayward AR, et al (2003) Decline in varicella-zoster virus (VZV)-specific cell-mediated immunity with increasing age and boosting with a high-dose VZV vaccine. J Infect Dis 188: 1336–1344. 14593591 10.1086/379048

[4] Berger R, Florent G, Just M (1981) Decrease of the lymphoproliferative response to varicella-zoster virus antigen in the aged. Infect Immun 32: 24–27. 6163722 10.1128/iai.32.1.24-27.1981 PMC350580

[5] Miller AE (1980) Selective decline in cellular immune response to varicella-zoster in the elderly. Neurology 30: 582–587. 6247671 10.1212/wnl.30.6.582

[6] Thomas SL, Hall AJ (2004) What does epidemiology tell us about risk factors for herpes zoster? Lancet Infect Dis 4: 26–33. 14720565 10.1016/s1473-3099(03)00857-0

[7] Bilcke J, Ogunjimi B, Marais C, de Smet F, Callens M, et al (2012) The health and economic burden of chickenpox and herpes zoster in Belgium. Epidemiol Infect 140: 2096–2109. 22230041 10.1017/S0950268811002640

[8] Levin MJ, Oxman MN, Zhang JH, Johnson GR, Stanley H, et al (2008) Varicella-Zoster Virus–Specific Immune Responses in Elderly Recipients of a Herpes Zoster Vaccine. The Journal of Infectious Diseases 197: 825–835. 18419349 10.1086/528696 PMC4014857

[9] Hope-Simpson RE (1965) The Nature of Herpes Zoster: A Long-Term Study and a New Hypothesis. Proc R Soc Med 58: 9–20. 14267505 10.1177/003591576505800106 PMC1898279

[10] Schuette MC, Hethcote HW (1999) Modeling the effects of varicella vaccination programs on the incidence of chickenpox and shingles. Bull Math Biol 61: 1031–1064. 17879870 10.1006/bulm.1999.0126

[11] Liberati A, Altman DG, Tetzlaff J, Mulrow C, Gotzsche PC, et al (2009) The PRISMA statement for reporting systematic reviews and meta-analyses of studies that evaluate health care interventions: explanation and elaboration. PLoS Med 6: e1000100. 19621070 10.1371/journal.pmed.1000100 PMC2707010

[12] Porru S, Campagna M, Arici C, Carta A, Placidi D, et al (2007) [Susceptibility to varicella-zoster, measles, rosacea and mumps among health care workers in a Northern Italy hospital]. G Ital Med Lav Ergon 29: 407–409. 18409748

[13] Brisson M, Gay NJ, Edmunds WJ, Andrews NJ (2002) Exposure to varicella boosts immunity to herpes-zoster: implications for mass vaccination against chickenpox. Vaccine 20: 2500–2507. 12057605 10.1016/s0264-410x(02)00180-9

[14] Mullooly JP, Riedlinger K, Chun C, Weinmann S, Houston H (2005) Incidence of herpes zoster, 1997–2002. Epidemiol Infect 133: 245–253. 15816149 10.1017/s095026880400281x PMC2870243

[15] Yih WK, Brooks DR, Lett SM, Jumaan AO, Zhang Z, et al (2005) The incidence of varicella and herpes zoster in Massachusetts as measured by the Behavioral Risk Factor Surveillance System (BRFSS) during a period of increasing varicella vaccine coverage, 1998–2003. BMC Public Health 5: 68. 15960856 10.1186/1471-2458-5-68 PMC1177968

[16] Jumaan AO, Yu O, Jackson LA, Bohlke K, Galil K, et al (2005) Incidence of herpes zoster, before and after varicella-vaccination-associated decreases in the incidence of varicella, 1992–2002. J Infect Dis 191: 2002–2007. 15897984 10.1086/430325

[17] Patel MS, Gebremariam A, Davis MM (2008) Herpes zoster-related hospitalizations and expenditures before and after introduction of the varicella vaccine in the United States. Infect Control Hosp Epidemiol 29: 1157–1163. 18999945 10.1086/591975

[18] Rimland D, Moanna A (2010) Increasing incidence of herpes zoster among Veterans. Clin Infect Dis 50: 1000–1005. 20178416 10.1086/651078

[19] Carville KS, Riddell MA, Kelly HA (2010) A decline in varicella but an uncertain impact on zoster following varicella vaccination in Victoria, Australia. Vaccine 28: 2532–2538. 20117265 10.1016/j.vaccine.2010.01.036

[20] Grant KA, Carville KS, Kelly HA (2010) Evidence of increasing frequency of herpes zoster management in Australian general practice since the introduction of a varicella vaccine. Med J Australia 193: 483–483. 20955129 10.5694/j.1326-5377.2010.tb04009.x

[21] Carville KS, Grant KA, Kelly HA (2012) Herpes zoster in Australia. Epidemiol Infect 140: 599–600; author reply 600–591. 10.1017/S095026881100149X 21849096

[22] Nelson MR, Britt HC, Harrison CM (2010) Evidence of increasing frequency of herpes zoster management in Australian general practice since the introduction of a varicella vaccine. Med J Australia 193: 110–113. 20642419

[23] Heywood AE, Macartney KK (2011) How can we better understand trends in varicella zoster virus-related disease epidemiology? Med J Australia 194: 268–269. 10.5694/j.1326-5377.2011.tb02965.x 21382005

[24] Jardine A, Conaty SJ, Vally H (2011) Herpes zoster in Australia: evidence of increase in incidence in adults attributable to varicella immunization? Epidemiol Infect 139: 658–665. 20727248 10.1017/S0950268810001949

[25] Tanuseputro P, Zagorski B, Chan KJ, Kwong JC (2011) Population-based incidence of herpes zoster after introduction of a publicly funded varicella vaccination program. Vaccine 29: 8580–8584. 21939721 10.1016/j.vaccine.2011.09.024

[26] Leung J, Harpaz R, Molinari NA, Jumaan A, Zhou FJ (2011) Herpes Zoster Incidence Among Insured Persons in the United States, 1993–2006: Evaluation of Impact of Varicella Vaccination. Clin Infect Dis 52: 332–340. 21217180 10.1093/cid/ciq077

[27] Chao DY, Chien YZ, Yeh YP, Hsu PS, Lian IB (2012) The incidence of varicella and herpes zoster in Taiwan during a period of increasing varicella vaccine coverage, 2000–2008. Epidemiol Infect 140: 1131–1140. 21906410 10.1017/S0950268811001786

[28] Ogunjimi B, Hens N, Goeyvaerts N, Aerts M, Van Damme P, et al (2009) Using empirical social contact data to model person to person infectious disease transmission: an illustration for varicella. Math Biosci 218: 80–87. 19174173 10.1016/j.mbs.2008.12.009

[29] Garnett GP, Grenfell BT (1992) The epidemiology of varicella-zoster virus infections: the influence of varicella on the prevalence of herpes zoster. Epidemiol Infect 108: 513–528. 1318219 10.1017/s0950268800050019 PMC2272211

[30] Brisson M, Edmunds WJ, Gay NJ, Law B, De Serres G (2000) Modelling the impact of immunization on the epidemiology of varicella zoster virus. Epidemiol Infect 125: 651–669. 11218215 10.1017/s0950268800004714 PMC2869648

[31] Bonmarin I, Santa-Olalla P, Levy-Bruhl D (2008) [Modelling the impact of vaccination on the epidemiology of varicella zoster virus]. Rev Epidemiol Sante Publique 56: 323–331. 18951741 10.1016/j.respe.2008.07.087

[32] Brisson M, Melkonyan G, Drolet M, De Serres G, Thibeault R, et al (2010) Modeling the impact of one- and two-dose varicella vaccination on the epidemiology of varicella and zoster. Vaccine 28: 3385–3397. 20199763 10.1016/j.vaccine.2010.02.079

[33] Oxman MN, Levin MJ, Johnson GR, Schmader KE, Straus SE, et al (2005) A vaccine to prevent herpes zoster and postherpetic neuralgia in older adults. N Engl J Med 352: 2271–2284. 15930418 10.1056/NEJMoa051016

[34] van Hoek AJ, Melegaro A, Zagheni E, Edmunds WJ, Gay N (2011) Modelling the impact of a combined varicella and zoster vaccination programme on the epidemiology of varicella zoster virus in England. Vaccine 29: 2411–2420. 21277405 10.1016/j.vaccine.2011.01.037

[35] Karhunen M, Leino T, Salo H, Davidkin I, Kilpi T, et al (2010) Modelling the impact of varicella vaccination on varicella and zoster. Epidemiol Infect 138: 469–481. 19796447 10.1017/S0950268809990768

[36] Solomon BA, Kaporis AG, Glass AT, Simon SI, Baldwin HE (1998) Lasting immunity to varicella in doctors study (LIVID study). J Am Acad Dermatol 38: 763–765. 9591824 10.1016/s0190-9622(98)70207-5

[37] Thomas SL, Wheeler JG, Hall AJ (2002) Contacts with varicella or with children and protection against herpes zoster in adults: a case-control study. Lancet 360: 678–682. 12241874 10.1016/S0140-6736(02)09837-9

[38] Chaves SS, Santibanez TA, Gargiullo P, Guris D (2007) Chickenpox exposure and herpes zoster disease incidence in older adults in the U.S. Public Health Rep. 122: 155–159. 10.1177/003335490712200204 PMC1820439 17357357

[39] Donahue JG, Kieke BA, Gargiullo PM, Jumaan AO, Berger NR, et al (2010) Herpes zoster and exposure to the varicella zoster virus in an era of varicella vaccination. Am J Public Health 100: 1116–1122. 20075320 10.2105/AJPH.2009.160002 PMC2866606

[40] Wu CY, Hu HY, Huang N, Pu CY, Shen HC, et al (2010) Do the health-care workers gain protection against herpes zoster infection? A 6-year population-based study in Taiwan. J Dermatol 37: 463–470. 20536652 10.1111/j.1346-8138.2010.00804.x

[41] Salleras M, Dominguez A, Soldevila N, Prat A, Garrido P, et al (2011) Contacts with children and young people and adult risk of suffering herpes zoster. Vaccine 29: 7602–7605. 21889558 10.1016/j.vaccine.2011.08.023

[42] Gaillat J, Gajdos V, Launay O, Malvy D, Demoures B, et al (2011) Does monastic life predispose to the risk of Saint Anthony's fire (herpes zoster)? Clin Infect Dis 53: 405–410. 21844022 10.1093/cid/cir436

[43] Gaillat J, Soubeyrand B, Malvy D, Caulin E, Launay O, et al (2012) Zoster in Monasteries: Some Clarification Needed Reply. Clin Infect Dis 54: 306–U319. 10.1093/cid/cir841 22198994

[44] Ogunjimi B, Van Damme P, Beutels P (2012) Zoster in monasteries: some clarification needed. Clin Infect Dis 54: 305–306; author reply 306–307. 10.1093/cid/cir841 22198994

[45] Lasserre A, Blaizeau F, Gorwood P, Bloch K, Chauvin P, et al (2012) Herpes zoster: family history and psychological stress-case-control study. J Clin Virol 55: 153–157. 22824229 10.1016/j.jcv.2012.06.020

[46] Arvin AM, Koropchak CM, Wittek AE (1983) Immunologic evidence of reinfection with varicella-zoster virus. J Infect Dis 148: 200–205. 6310001 10.1093/infdis/148.2.200

[47] Gershon AA, Steinberg SP (1990) Live attenuated varicella vaccine: protection in healthy adults compared with leukemic children. National Institute of Allergy and Infectious Diseases Varicella Vaccine Collaborative Study Group. J Infect Dis 161: 661–666. 2156941 10.1093/infdis/161.4.661

[48] Vossen MT, Gent MR, Weel JF, de Jong MD, van Lier RA, et al (2004) Development of virus-specific CD4+ T cells on reexposure to Varicella-Zoster virus. J Infect Dis 190: 72–82. 15195245 10.1086/421277

[49] Ogunjimi B, Smits E, Hens N, Hens A, Lenders K, et al (2011) Exploring the impact of exposure to primary varicella in children on varicella-zoster virus immunity of parents. Viral Immunol 24: 151–157. 21449725 10.1089/vim.2010.0031

[50] Lenders K, Ogunjimi B, Beutels P, Hens N, Van Damme P, et al (2010) The effect of apoptotic cells on virus-specific immune responses detected using IFN-gamma ELISPOT. J Immunol Methods 357: 51–54. 20214906 10.1016/j.jim.2010.03.001

[51] Smith JG, Liu X, Kaufhold RM, Clair J, Caulfield MJ (2001) Development and validation of a gamma interferon ELISPOT assay for quantitation of cellular immune responses to varicella-zoster virus. Clin Diagn Lab Immunol 8: 871–879. 11527795 10.1128/CDLI.8.5.871-879.2001 PMC96163

[52] Gershon AA, Steinberg SP, Borkowsky W, Lennette D, Lennette E (1982) IgM to varicella-zoster virus: demonstration in patients with and without clinical zoster. Pediatr Infect Dis 1: 164–167. 6292875 10.1097/00006454-198205000-00007

[53] Terada K, Kawano S, Yoshihiro K, Morita T (1993) Proliferative response to varicella-zoster virus is inverse related to development of high levels of varicella-zoster virus specific IgG antibodies. Scand J Infect Dis 25: 775–778. 8052820 10.3109/00365549309008578

[54] Terada K, Niizuma T, Yagi Y, Miyashima H, Kataoka N, et al (2000) Low induction of varicella-zoster virus-specific secretory IgA antibody after vaccination. J Med Virol 62: 46–51. 10935988

[55] Yavuz T, Ozdemir I, Sencan I, Arbak P, Behcet M, et al (2005) Seroprevalence of varicella, measles and hepatitis B among female health care workers of childbearing age. Jpn J Infect Dis 58: 383–386. 16377874

[56] Saadatian-Elahi M, Mekki Y, Del Signore C, Lina B, Derrough T, et al (2007) Seroprevalence of varicella antibodies among pregnant women in Lyon-France. Eur J Epidemiol 22: 405–409. 17534728 10.1007/s10654-007-9136-z

[57] Valdarchi C, Farchi F, Dorrucci M, De Michetti F, Paparella C, et al (2008) Epidemiological investigation of a varicella outbreak in an Italian prison. Scand J Infect Dis 40: 943–945. 18720259 10.1080/00365540802308449

[58] Toyama N, Shiraki K (2009) Epidemiology of herpes zoster and its relationship to varicella in Japan: A 10-year survey of 48,388 herpes zoster cases in Miyazaki prefecture. J Med Virol 81: 2053–2058. 19856466 10.1002/jmv.21599

[59] Goeyvaerts N, Hens N, Ogunjimi B, Aerts M, Shkedy Z, et al (2010) Estimating infectious disease parameters from data on social contacts and serological status. Journal of the Royal Statistical Society Series C-Applied Statistics 59: 255–277.

[60] Levin MJ, Murray M, Zerbe GO, White CJ, Hayward AR (1994) Immune responses of elderly persons 4 years after receiving a live attenuated varicella vaccine. J Infect Dis 170: 522–526. 8077709 10.1093/infdis/170.3.522

[61] Ogunjimi B, Theeten H, Hens N, Beutels P (submitted) Serology indicates Cytomegalovirus infection is instrumental in Varicella-Zoster Virus reactivation. 10.1002/jmv.23749 24037981

