# Supplementary material for: Herpes Zoster Risk Reduction through Exposure to Chickenpox Patients: A Systematic Multidisciplinary Review
Source: PLoS One. 2013 Jun 21;8(6):e66485. doi: 10.1371/journal.pone.0066485 (PMC3689818; doi:10.1371/journal.pone.0066485)
Supplement: Table S1 — Inclusion algorithm. (DOC) [file pone.0066485.s001.doc]

Supplementary Table S1. Inclusion algorithm

1. Original research article or multiple cases (no reviews, guidelines, recommendations, etc.) with VZV as one of the study endpoints
2. Exclusion if limited solely to
   1. HZ incidence, risk factors or immunological responses in individuals vaccinated against VZV or with clinical signs of VZV-related disease
   2. Use of antivirals or VZV immunoglobulins
   3. Immunosuppressive states, e.g. HIV, cancer, lupus, etc.
   4. Children (<18y)
   5. HZ incidence reported by only one GP practice or only one hospital
3. Screening of title/abstracts categorizes references in
   1. Immunological or clinical
      1. Inclusion if
         1. evidence of re-exposure to CP (not HZ) AND assessment of
            1. VZV-specific immunity
            2. or HZ incidence
         2. studies should either be
            1. longitudinal post-exposure
            2. or single time point

with control/reference group

OR different exposure rates between groups

- 1. (sero-)epidemiological or mathematical modelling
     1. (sero-)epidemiological
        1. Inclusion*
           1. when comparing HZ incidence or VZV-serology§ between groups with a different explicit exposure to CP
           2. or when comparing HZ incidence or VZV-serology§ between groups with a different implicit exposure to CP (e.g. living with children or other items in questionnaires)
           3. or when examining the population effect of childhood CP vaccination on HZ incidence or VZV-serology§
     2. mathematical modeling
        1. Inclusion
           1. when existence of exogenous boosting is contrasted to the absence of exogenous boosting (so not only used as input parameter)
           2. or when comparing correlation between CP incidence and HZ incidence using statistical techniques (e.g. time series)*VZV* varicella-zoster virus; *HZ* herpes zoster; *GP* general practitioner; *CP* chickenpox.

*Studies with co-morbidities are categorized as ‘immunological or clinical’.

§Not only dichotomous but using quantitative titers.
