# Supplementary material for: Herpes Zoster Risk Reduction through Exposure to Chickenpox Patients: A Systematic Multidisciplinary Review
Source: PLoS One. 2013 Jun 21;8(6):e66485. doi: 10.1371/journal.pone.0066485 (PMC3689818; doi:10.1371/journal.pone.0066485)
Supplement: Table S2 — Grading of different study designs. (DOC) [file pone.0066485.s002.doc]

Supplementary Table S2. Grading of different study designs

| **Study design*** | **Grade** |
| --- | --- |
| Two otherwise comparable groups only differing in exposure to CP. Group A is uniquely exposed to CP at a given time point, whereas group B is not exposed to CP at any time in the observational period. Ideally, HZ incidences should be compared between the two groups, whilst controlling for potential boosting by HZ. | A+ |
| Two otherwise comparable groups with CP circulating in one group, but not or less in the other. Ideally, the cohort's HZ incidences should be compared with correction for HZ exposure. | A |
| Within one geographical region HZ incidence pre- and post-CP-vaccination are compared, whilst controlling for confounding variables such as an ageing demography | A |
| Accepting HZ only occurs or is more likely when CMI is low, implies that CMI boosting delays HZ occurrence. Therefore a prospective immunological study in which CMI is monitored in a boosted versus a control group allows estimating the duration of the boosting effect and thus the effect of CP exogenous boosting. | A |
| Case-control study with explicit CP exposure (+ HZ exposure or other confounding factors) within a given time-frame (e.g. 5 and 10 years) to estimate the OR/RR of exposure. | B |
| HZ incidence is compared between groups, of which can be assumed that their frequency of exposure to CP differs (e.g. pediatricians vs. call center employees, living with children), whilst controlling for confounding variables. | B |
| Two not necessarily comparable groups with differing CP circulation for which HZ incidence is explicitly compared after controlling for several variables. | B |
| Mathematical models with and without boosting by CP are compared in how they fit HZ incidence rates by age and/or time since CP vaccination. The fitting process preferably involves goodness-of-fit criteria. | C |
| Two otherwise comparable groups with one having a higher probability to be exposed to CP (for e.g. by children living in the household or working as a pediatrician). Again, the groups’ HZ incidences will be compared, whilst ideally controlling for confounding variables. | C |
| CMI for groups differing in assumed level of exposure to CP is cross-sectionally compared. | C |
| CMI for a group in which a high level of exposure to CP/HZ is expected is longitudinally followed to observe the duration of boosting. | C |
| Case-control study with higher cumulative probability of exposure to CP within a given time-frame (for e.g. living with children), whilst ideally controlling for confounding variables. | C |
| HMI for groups differing in assumed level of exposure to CP is cross-sectionally compared (for e.g. serological assessment of health care workers). | D |
| HMI for a group in which a high level of exposure to CP/HZ is expected is longitudinally followed to observe duration of boosting. | D |
| Re-exposure study with VZV-specific IgG titers before re-exposure compared to after or to titers from control group | D |
| Re-exposure study with VZV-specific IgG response after re-exposure analyzed as a function of the number of previous CP exposures | D |
| Re-exposure study with observation of VZV-specific IgM after re-exposure, only if history of CP | D |

*CP* chickenpox; *HZ* herpes zoster; *CMI* cellular mediated immunity; *HMI* humoral mediated immunity.

*In general a quantitative approach should be undertaken
