# Supplementary material for: Herpes Zoster Risk Reduction through Exposure to Chickenpox Patients: A Systematic Multidisciplinary Review
Source: PLoS One. 2013 Jun 21;8(6):e66485. doi: 10.1371/journal.pone.0066485 (PMC3689818; doi:10.1371/journal.pone.0066485)
Supplement: Table S3 — Potential biases identified for each included study. (DOC) [file pone.0066485.s003.doc]

Supplementary Table S3. Potential biases identified for each included study

| Reference | Quality* | Potential bias |
| --- | --- | --- |
| **Observational studies on HZ incidence following widespread chickenpox vaccination** | | |
| Mullooly et al (2005) | M | No adequate comparison (in regard to same region) between pre-vaccination and post-vaccination Limited account for immunosuppressive or underlying diseases Possibly not enough time between CP decrease and HZ observations HMO database: potential selection bias  HZ incidence estimation does not take decrease (through VZV vaccination) in naturally infected children into account |
| Yih et al (2005) | M | Telephone survey based on recollection (without validation): uncertain diagnosis No elaborate pre-vaccination data so difficult to interpret causality  No control or assessment for environmental changes (immunosuppression) Low response rates  No account for immunosuppressive or underlying diseases HZ incidence estimation doesn’t take decrease in natural infected children in account |
| Jumaan et al (2005) | M | CP decrease only started in 1999, so we can at most only expect to see a minor effect on HZ  Relatively low coverage could lead to a masking of the effect of exogenous boosting HMO registration could lead to bias Possible underreporting of CP Positive predictive value of diagnosis of varicella decreased over time, ranging from an average of 85% during 1992–1994 (before vaccination) to 53% during 1999–2000 Overall incidence estimation doesn’t take decrease in naturally infected children in account |
| Patel et al (2008) | L | Hospitalizations combined both primary and secondary HZ, so an increase in underlying diseases (and registration changes) could perturb the analysis; as Harpaz et al discuss the expansion in participating states throughout the study years could further increase a possible bias created by including secondary HZ diagnoses No trend or regression analyses Hospitalization rates are a limited proxy to examine overall HZ occurrence Limited time frame since CP vaccination HZ incidence estimation doesn’t take decrease in naturally infected children in account |
| Rimland et al (2010) | M | Veterans could form a biased group No data on CP incidence during observation period No pre-vaccination data available Use of steroids or TNF-alpha inhibitors was not considered immunocompromising The authors noted that the study was limited to the inclusion of HZ cases registered by the VA and that diagnoses from other medical centers were thus not included; potentially, annual changes in health care access could have influenced the registration of HZ Unclear whether immunocompetent-specific analysis also included age-standardization |
| Carville et al (2010) | M | MMDS possibly does not offer a representative registration of overall CP or HZ incidence Limited time frame since introduction CP vaccination MMDS data do not account for immunosuppressive or underlying diseases HZ incidence estimation doesn’t take decrease in naturally infected children in account |
| Nelson et al (2010) | L | Only 1 pre-vaccination year was examined, so difficult to assess any pre-vaccination trends Rates are calculated per 1000 consultations; this could lead to biases Uncertain whether the higher HZ incidence in 1998 was taken into account for the linear regression Limited time frame since introduction CP vaccination No age-standardization; Heywood et al also noted that a crude HZ incidence increase was noted in the years before CP vaccination which was due to an ageing population No account for immunosuppressive or underlying diseases HZ incidence estimation doesn’t take decrease in naturally infected children in account |
| Grant et al (2010) | L | Only 1 pre-vaccination year was examined, so difficult to assess any pre-vaccination trends Rates are calculated per 1000 consultations; this could lead to biases No age-standardization for overall HZ incidence calculation No account for immunosuppressive or underlying diseases HZ incidence estimation doesn’t take decrease in naturally infected children in account |
| Jardine et al (2011) | L | Use of antivirals is normally only indicated within a limited time frame since HZ onset and could this be not used when HZ was diagnosed too late; however, a correction would potentiate the increase in HZ; furthermore, prescriptions for antivirals aren't a good proxy for the unique incidence of HZ; they also can be used for other diseases (as noted by Carville et al ); the prescribing could be influenced by changing practice guidelines or habits No data in regard to CP occurrence No sufficient pre-vaccination data available No account for immunosuppressive or underlying diseases HZ incidence estimation doesn’t take decrease in naturally infected children in account |
| Carville et al (2012) | L | MMDS possible does not offer a representative registration of overall CP or HZ incidence No data in regard to CP occurrence No sufficient pre-vaccination data available No account for immunosuppressive or underlying diseases HZ incidence estimation doesn’t take decrease in naturally infected children in account |
| Tanuseputro et al (2011) | M | No data in regard to CP incidence Limited time frame since introduction CP vaccination Not a sufficient account for immunosuppressive or underlying diseases HZ incidence estimation doesn’t take decrease in naturally infected children in account |
| Leung et al (2011) | H | Major demographic shift in database where 65+ went from 1% at the beginning to 10-16% leading to possible biases Uncertainty about level of vaccination coverage, also no addition of CP incidences  No information in regard to the actual CP incidences in the states with high and low CP vaccine coverage No idea for timeframe of having children dependent, nor any idea whether dependency means living together in HH HZ incidence estimation doesn’t take decrease in naturally infected children in account |
| Chao et al (2012) | H | No full dataset from pre-CP-vaccination Limited time frame since introduction CP vaccination The increase in HZ could coincide with the increasing use of registration; this could both explain the overall increase in HZ, but could also explain the pre-vaccination increase HZ incidence estimation doesn’t take decrease in naturally infected children in account |
| **Prospective longitudinal studies on VZV-immunity post exposure** | | |
| Arvin et al (1983) | L | No virological confirmation of VZV in contacts Arbitrary definition of positive vs. negative Only (young) women with children in HH, no information concerning exposure or matching in CO Old, less accurate techniques to detect immunity: only indication of cell proliferation and no effector function, no phenotyping, etc. Only one month between sampling points |
| Gershon et al (1990) | L | Unclear whether parents were exposed to breakthrough CP or natural CP No CO, but authors refer to previous study where 2% of seropositives had FAMA titers ≥ 1:64 No statistical calculations No analysis in regard to timing Less quantitative antibody measurement technique |
| Vossen et al (2004) | M | No virological confirmation of VZV in contacts Arbitrary definition of positive vs. negative No quantitative assessment on duration of boosting  No ELISPOT data presented No longitudinal CO Use of cell lysate in cryopreserved samples could bias interpretation of assays, particularly the CD8-response No known matching between RE and CO, relatively young age RE with no information for CO No assessment (for e.g. viability) of longitudinal bias due to cryopreservation |
| Ogunjimi et al (2011) | M | No virological confirmation of VZV in contacts Possible bias due to long term freezing (see viability differences), could explain dip in ELISPOT response at 1mo and ELISPOT values not being higher in RE than young CO Use of cell lysate in cryopreserved samples could bias interpretation of assays No cell phenotyping when using ELISPOT Relatively young age of RE No long-term extrapolation performed No longitudinal CO, also cryopreservation time differed substantially between RE and CO |
| **Mathematical modeling studies** | | |
| Garnett & Grenfell (1992) | M | CP notifiable, but HZ not Boosting had continuous exponential time function without use of thresholds and without examination of other functional forms Use of WAIFW matrices could lead to biases No quantitative comparison between scenarios of boosting |
| Brisson et al (2000) | M | Use of WAIFW matrices could lead to biases No comparison between scenarios with or without boosting Circularity due to fitting the reactivation rate to HZ incidence data No biological support for existence of S_boost_ compartment or for postulation of to be 2 or 20 years (also unclear what was used in estimation) |
| Brisson et al (2002) | M | In total 11 parameters to be estimated from 364 data points: no sensitivity analysis performed; although a CI was given for  no CI was given for most parameters fitted Possible trade offs between rates. Definition of ’s could lead to biases Assumption of 100% boosting No direct exposure to CP modeled, but it was assumed that living with children was a good proxy for exposure to CP  Even if the indirect exposure modeling would not be problematic, the definition of the ’s and the reactivation rate could lead to an erroneous estimation of  No biological support for existence S_boost_ compartment |
| Bonmarin et al (2008) | L | Use of WAIFW matrices could lead to biases Definition and derivation of WAIFW matrices were not mathematically presented No comparison between scenarios with or without boosting No biological support for existence of S_boost_ compartment or for postulation of to be 20 years  Assumption of 100% boosting Not clear how parameters were adjusted from Brisson et al to the French data Limited comparison between simulated and observed data |
| Brisson et al (2010) | M | Same comments as for Brisson et al (with the exception of 100% boosting assumption) No comparison with no boosting scenario concerning goodness of fit Circularity due to fitting the reactivation rate to HZ incidence data Appraisal of qualitative fit to USA post-vaccination data is dependent on CP vaccination components of model Use of empirical contact matrix structure from averaged Europe and not from Canada or USA (also unclear how other parameters are estimated for the comparison with the USA post-vaccination data) |
| Van Hoek et al (2011) | M | Same comments as Brisson et al  No comparison with no boosting scenario concerning goodness of fit Circularity due to fitting the reactivation rate to HZ incidence data No biological support for existence of S_boost_ compartment  Postulation of σ to be 20 years  Infectious period HZ 7 days |
| Karhunen et al (2010) | M | Ambulatory care data, incidence of HZ underestimated by 10% Infectious period HZ 7 days  Threshold for age-induced waning assumes in this model that for all ages before threshold only boosting can explain differences in HZ incidence between age groups; however, the HZ incidence data are quite constant between 10-45y, while the distribution of time since the last exposure is not; also, before the age threshold, the boosting parameter will be estimated by data points from (1) individuals in age groups with low HZ incidence and low seroprevalence leading to 0y since boosting (0-10y) and (2) individuals in age groups with higher HZ incidence (>90% seroprevalence) and being recently boosted; placing time since first infection and boosting at the same level thus perturbs the estimation of boosting; these errors will lead to a bias in the estimation of the basal reactivation rate and and consequently also for the age-waning rate |
| **Epidemiological risk factor studies** | | |
| Solomon et al (1998) | L | No multivariate analysis controlling for gender, HH VZV exposure or age  No analysis focusing on number of annual VZV contacts  Low response rate, particularly for psychiatrists (13%): prone to bias No specification whether CP and HZ exposure |
| Thomas et al (2002) | H | Inclusion for multivariate analysis based on univariate P value < 0.2  Hierarchical multivariate approach could lead to over-exclusion No sensitivity analysis related to the recollection time of 10y  No information on duration of exposure |
| Brisson et al (2002) | H | Cross sectional data; no information on previous living with child in household |
| Chaves et al (2007) | M | Post-vaccination study: breakthrough CP could lead to lower potency of transmission & lower CP incidence, and therefore increased risk of misdiagnosis  HZ boosting could mask effect of CP  Limited data analysis  Telephone survey bias Questionable high HZ incidence (19/1000PY when ≥ 65y) |
| Donahue et al (2010) | M | Post-vaccination study: breakthrough CP could lead to lower potency of transmission & lower CP incidence, and therefore increased risk of misdiagnosis  Cases participating were more likely older and female (both increasing risk of HZ)  Limited data analysis  Telephone survey bias Rural population only Relative long time period between recall and actual HZ |
| Wu et al (2010) | M | Sparse data: only 7 cases for 168 dermatologists & pediatricians High HZ incidence (11.5-15.2/1000PY) at young age (20-39y) in dermatologists & pediatricians Unclear influence of CP vaccination in Taiwan (CP vaccine available since 1997 and universal program since 2004) |
| Salleras et al (2011) | M | Recall time of 10y  Although the contact data were separately per setting of contact (within household and without household) only aggregated total contact hours during the past 10 years were used for the analysis |
| Gaillat et al (2011) | M | See discussion  HZ incidence overall higher in females, 42% females in CMO vs. 52% in GP, but no prior prove of sufficient sample size  Power calculations are not based on a priori estimations of CP exposure rates amongst GP  No control for HZ exposure (particularly important for groups living in small congregants) Exclusion of individuals with HZ before entering (3% from original CMO) were excluded without similar exclusion in GP, could lead to underestimation of exogenous boosting effect No definition of 'regular' contact with children More diseases amongst CMO when having acute onset of HZ |
| Lasserre et al (2012) | H | HZ only clinically defined |
| **Other studies** | | |
| Gershon et al (1982) | M | No timing after re-exposure No information in regard to age-gender matching No statistical comparisons |
| Terada et al (1993) | M | Limitations of responder cell frequency assay (no differentiation possible) No matching of gender or age No statistical analysis on serology |
| Terada et al (2000) | L | No age or gender matching  No information about ages Only significance presented when comparing with children with CP 2 years ago |
| Yavuz et al (2005) | L | Health-care workers are on average 5y younger than controls, thus ageing (or longer time since infection) could explain the results Do data concerning type of VZV exposure |
| Saadation-Elahi et al (2007) | L | Uncertain whether pregnant women are sufficiently representative  Statistics without adjustment for confounding variables: more children could also mean older women and this could thus mask a rise in IgG. Better to perform multiple regression |
| Valdarchi et al (2008) | L | Some participants had HIV No time description Only seroprevalence |
| Toyama et al (2009) | L | No estimation of missed HZ cases consulting GPs or other medical doctors No statistical analysis (e.g. time series analysis) between CP and HZ incidence Possibility of bias due to breakthrough infections No information in regard to CP survey method |

*CP* chickenpox; *HZ* herpes zoster; *MMDS* Melbourne Medical Deputising Service; *HH* household; *VZV* varicella-zoster virus; *CMO* contemplative monastic orders; *GP* general practitioners; *ELISPOT* enzyme-linked immunosorbent spot; *CO* control group; *RE* re-exposed; *WAIFW* who-acquires-infection-from-whom.

*H=High: the quality of methods used in this paper permits the results, within the scope of the study design, to be interpreted with at the most a few remarks. M=Medium: the quality of methods used in this paper permits the results, within the scope of the study design, to be interpreted, but with some caution. L=Low: the quality of methods used in this paper urges the reader to interpret the results, even within the scope of the study design, with sufficient caution.

References

1. Mullooly JP, Riedlinger K, Chun C, Weinmann S, Houston H (2005) Incidence of herpes zoster, 1997-2002. Epidemiol Infect 133: 245-253.

2. Yih WK, Brooks DR, Lett SM, Jumaan AO, Zhang Z, et al. (2005) The incidence of varicella and herpes zoster in Massachusetts as measured by the Behavioral Risk Factor Surveillance System (BRFSS) during a period of increasing varicella vaccine coverage, 1998-2003. BMC Public Health 5: 68.

3. Jumaan AO, Yu O, Jackson LA, Bohlke K, Galil K, et al. (2005) Incidence of herpes zoster, before and after varicella-vaccination-associated decreases in the incidence of varicella, 1992-2002. J Infect Dis 191: 2002-2007.

4. Patel MS, Gebremariam A, Davis MM (2008) Herpes zoster-related hospitalizations and expenditures before and after introduction of the varicella vaccine in the United States. Infect Control Hosp Epidemiol 29: 1157-1163.

5. Harpaz R, Yawn BP (2009) Trends in rates of herpes zoster-related hospitalizations: are they real, are they costly, and are they linked to varicella vaccination? Infect Control Hosp Epidemiol 30: 495-496; author reply 496-497.

6. Rimland D, Moanna A (2010) Increasing incidence of herpes zoster among Veterans. Clin Infect Dis 50: 1000-1005.

7. Carville KS, Riddell MA, Kelly HA (2010) A decline in varicella but an uncertain impact on zoster following varicella vaccination in Victoria, Australia. Vaccine 28: 2532-2538.

8. Nelson MR, Britt HC, Harrison CM (2010) Evidence of increasing frequency of herpes zoster management in Australian general practice since the introduction of a varicella vaccine. Med J Australia 193: 110-113.

9. Heywood AE, Macartney KK (2011) How can we better understand trends in varicella zoster virus-related disease epidemiology? Med J Australia 194: 268-269.

10. Grant KA, Carville KS, Kelly HA (2010) Evidence of increasing frequency of herpes zoster management in Australian general practice since the introduction of a varicella vaccine. Med J Australia 193: 483-483.

11. Jardine A, Conaty SJ, Vally H (2011) Herpes zoster in Australia: evidence of increase in incidence in adults attributable to varicella immunization? Epidemiol Infect 139: 658-665.

12. Carville KS, Grant KA, Kelly HA (2012) Herpes zoster in Australia. Epidemiol Infect 140: 599-600; author reply 600-591.

13. Tanuseputro P, Zagorski B, Chan KJ, Kwong JC (2011) Population-based incidence of herpes zoster after introduction of a publicly funded varicella vaccination program. Vaccine 29: 8580-8584.

14. Leung J, Harpaz R, Molinari NA, Jumaan A, Zhou FJ (2011) Herpes Zoster Incidence Among Insured Persons in the United States, 1993-2006: Evaluation of Impact of Varicella Vaccination. Clin Infect Dis 52: 332-340.

15. Chao DY, Chien YZ, Yeh YP, Hsu PS, Lian IB (2012) The incidence of varicella and herpes zoster in Taiwan during a period of increasing varicella vaccine coverage, 2000-2008. Epidemiol Infect 140: 1131-1140.

16. Arvin AM, Koropchak CM, Wittek AE (1983) Immunologic evidence of reinfection with varicella-zoster virus. J Infect Dis 148: 200-205.

17. Gershon AA, Steinberg SP (1990) Live attenuated varicella vaccine: protection in healthy adults compared with leukemic children. National Institute of Allergy and Infectious Diseases Varicella Vaccine Collaborative Study Group. J Infect Dis 161: 661-666.

18. Vossen MT, Gent MR, Weel JF, de Jong MD, van Lier RA, et al. (2004) Development of virus-specific CD4+ T cells on reexposure to Varicella-Zoster virus. J Infect Dis 190: 72-82.

19. Ogunjimi B, Smits E, Hens N, Hens A, Lenders K, et al. (2011) Exploring the impact of exposure to primary varicella in children on varicella-zoster virus immunity of parents. Viral Immunol 24: 151-157.

20. Garnett GP, Grenfell BT (1992) The epidemiology of varicella-zoster virus infections: the influence of varicella on the prevalence of herpes zoster. Epidemiol Infect 108: 513-528.

21. Brisson M, Edmunds WJ, Gay NJ, Law B, De Serres G (2000) Modelling the impact of immunization on the epidemiology of varicella zoster virus. Epidemiol Infect 125: 651-669.

22. Brisson M, Gay NJ, Edmunds WJ, Andrews NJ (2002) Exposure to varicella boosts immunity to herpes-zoster: implications for mass vaccination against chickenpox. Vaccine 20: 2500-2507.

23. Bonmarin I, Santa-Olalla P, Levy-Bruhl D (2008) [Modelling the impact of vaccination on the epidemiology of varicella zoster virus]. Rev Epidemiol Sante Publique 56: 323-331.

24. Brisson M, Melkonyan G, Drolet M, De Serres G, Thibeault R, et al. (2010) Modeling the impact of one- and two-dose varicella vaccination on the epidemiology of varicella and zoster. Vaccine 28: 3385-3397.

25. van Hoek AJ, Melegaro A, Zagheni E, Edmunds WJ, Gay N (2011) Modelling the impact of a combined varicella and zoster vaccination programme on the epidemiology of varicella zoster virus in England. Vaccine 29: 2411-2420.

26. Karhunen M, Leino T, Salo H, Davidkin I, Kilpi T, et al. (2010) Modelling the impact of varicella vaccination on varicella and zoster. Epidemiol Infect 138: 469-481.

27. Solomon BA, Kaporis AG, Glass AT, Simon SI, Baldwin HE (1998) Lasting immunity to varicella in doctors study (LIVID study). J Am Acad Dermatol 38: 763-765.

28. Thomas SL, Wheeler JG, Hall AJ (2002) Contacts with varicella or with children and protection against herpes zoster in adults: a case-control study. Lancet 360: 678-682.

29. Chaves SS, Santibanez TA, Gargiullo P, Guris D (2007) Chickenpox exposure and herpes zoster disease incidence in older adults in the U.S. Public Health Rep 122: 155-159.

30. Donahue JG, Kieke BA, Gargiullo PM, Jumaan AO, Berger NR, et al. (2010) Herpes zoster and exposure to the varicella zoster virus in an era of varicella vaccination. Am J Public Health 100: 1116-1122.

31. Wu CY, Hu HY, Huang N, Pu CY, Shen HC, et al. (2010) Do the health-care workers gain protection against herpes zoster infection? A 6-year population-based study in Taiwan. J Dermatol 37: 463-470.

32. Salleras M, Dominguez A, Soldevila N, Prat A, Garrido P, et al. (2011) Contacts with children and young people and adult risk of suffering herpes zoster. Vaccine 29: 7602-7605.

33. Gaillat J, Gajdos V, Launay O, Malvy D, Demoures B, et al. (2011) Does monastic life predispose to the risk of Saint Anthony's fire (herpes zoster)? Clin Infect Dis 53: 405-410.

34. Gaillat J, Soubeyrand B, Malvy D, Caulin E, Launay O, et al. (2012) Zoster in Monasteries: Some Clarification Needed Reply. Clin Infect Dis 54: 306-U319.

35. Ogunjimi B, Van Damme P, Beutels P (2012) Zoster in monasteries: some clarification needed. Clin Infect Dis 54: 305-306; author reply 306-307.

36. Lasserre A, Blaizeau F, Gorwood P, Bloch K, Chauvin P, et al. (2012) Herpes zoster: family history and psychological stress-case-control study. J Clin Virol 55: 153-157.

37. Gershon AA, Steinberg SP, Borkowsky W, Lennette D, Lennette E (1982) IgM to varicella-zoster virus: demonstration in patients with and without clinical zoster. Pediatr Infect Dis 1: 164-167.

38. Terada K, Kawano S, Yoshihiro K, Morita T (1993) Proliferative response to varicella-zoster virus is inverse related to development of high levels of varicella-zoster virus specific IgG antibodies. Scand J Infect Dis 25: 775-778.

39. Terada K, Niizuma T, Yagi Y, Miyashima H, Kataoka N, et al. (2000) Low induction of varicella-zoster virus-specific secretory IgA antibody after vaccination. J Med Virol 62: 46-51.

40. Yavuz T, Ozdemir I, Sencan I, Arbak P, Behcet M, et al. (2005) Seroprevalence of varicella, measles and hepatitis B among female health care workers of childbearing age. Jpn J Infect Dis 58: 383-386.

41. Saadatian-Elahi M, Mekki Y, Del Signore C, Lina B, Derrough T, et al. (2007) Seroprevalence of varicella antibodies among pregnant women in Lyon-France. Eur J Epidemiol 22: 405-409.

42. Valdarchi C, Farchi F, Dorrucci M, De Michetti F, Paparella C, et al. (2008) Epidemiological investigation of a varicella outbreak in an Italian prison. Scand J Infect Dis 40: 943-945.

43. Toyama N, Shiraki K (2009) Epidemiology of herpes zoster and its relationship to varicella in Japan: A 10-year survey of 48,388 herpes zoster cases in Miyazaki prefecture. J Med Virol 81: 2053-2058.
